# *Helicobacter pylori* multiplex serology and risk of non-cardia and cardia gastric cancer: a case-cohort study and meta-analysis

**DOI:** 10.1093/ije/dyad007

**Published:** 2023-03-13

**Authors:** Pang Yao, Christiana Kartsonaki, Julia Butt, Rima Jeske, Catherine de Martel, Martyn Plummer, Yu Guo, Sarah Clark, Robin G Walters, Yiping Chen, Daniel Avery, Jun Lv, Canqing Yu, Hao Wang, Michael Hill, Richard Peto, Liming Li, Tim Waterboer, Zhengming Chen, Iona Y Millwood, Ling Yang

**Affiliations:** Clinical Trial Service Unit & Epidemiological Studies Unit (CTSU), Nuffield Department of Population Health, University of Oxford, Oxford, UK; Clinical Trial Service Unit & Epidemiological Studies Unit (CTSU), Nuffield Department of Population Health, University of Oxford, Oxford, UK; Medical Research Council Population Health Research Unit (MRC PHRU), Nuffield Department of Population Health, University of Oxford, Oxford, UK; Infections and Cancer Epidemiology Division, German Cancer Research Center (DKFZ), Heidelberg, Germany; Infections and Cancer Epidemiology Division, German Cancer Research Center (DKFZ), Heidelberg, Germany; Early Detection, Prevention and Infections Branch, International Agency for Research on Cancer, Lyon, France; Department of Statistics, University of Warwick, Coventry, UK; National Center for Cardiovascular Diseases, Fuwai Hospital, Chinese Academy of Medical Sciences, Beijing, China; Clinical Trial Service Unit & Epidemiological Studies Unit (CTSU), Nuffield Department of Population Health, University of Oxford, Oxford, UK; Medical Research Council Population Health Research Unit (MRC PHRU), Nuffield Department of Population Health, University of Oxford, Oxford, UK; Clinical Trial Service Unit & Epidemiological Studies Unit (CTSU), Nuffield Department of Population Health, University of Oxford, Oxford, UK; Medical Research Council Population Health Research Unit (MRC PHRU), Nuffield Department of Population Health, University of Oxford, Oxford, UK; Clinical Trial Service Unit & Epidemiological Studies Unit (CTSU), Nuffield Department of Population Health, University of Oxford, Oxford, UK; Medical Research Council Population Health Research Unit (MRC PHRU), Nuffield Department of Population Health, University of Oxford, Oxford, UK; Clinical Trial Service Unit & Epidemiological Studies Unit (CTSU), Nuffield Department of Population Health, University of Oxford, Oxford, UK; Department of Epidemiology and Biostatistics, School of Public Health, Peking University, Beijing, China; Center for Public Health and Epidemic Preparedness & Response, Peking University, Beijing, China; Department of Epidemiology and Biostatistics, School of Public Health, Peking University, Beijing, China; Center for Public Health and Epidemic Preparedness & Response, Peking University, Beijing, China; NCDs Prevention and Control Department, Zhejiang CDC, Zhejiang, China; Clinical Trial Service Unit & Epidemiological Studies Unit (CTSU), Nuffield Department of Population Health, University of Oxford, Oxford, UK; Medical Research Council Population Health Research Unit (MRC PHRU), Nuffield Department of Population Health, University of Oxford, Oxford, UK; Clinical Trial Service Unit & Epidemiological Studies Unit (CTSU), Nuffield Department of Population Health, University of Oxford, Oxford, UK; Department of Epidemiology and Biostatistics, School of Public Health, Peking University, Beijing, China; Center for Public Health and Epidemic Preparedness & Response, Peking University, Beijing, China; Infections and Cancer Epidemiology Division, German Cancer Research Center (DKFZ), Heidelberg, Germany; Clinical Trial Service Unit & Epidemiological Studies Unit (CTSU), Nuffield Department of Population Health, University of Oxford, Oxford, UK; Medical Research Council Population Health Research Unit (MRC PHRU), Nuffield Department of Population Health, University of Oxford, Oxford, UK; Clinical Trial Service Unit & Epidemiological Studies Unit (CTSU), Nuffield Department of Population Health, University of Oxford, Oxford, UK; Medical Research Council Population Health Research Unit (MRC PHRU), Nuffield Department of Population Health, University of Oxford, Oxford, UK; Clinical Trial Service Unit & Epidemiological Studies Unit (CTSU), Nuffield Department of Population Health, University of Oxford, Oxford, UK; Medical Research Council Population Health Research Unit (MRC PHRU), Nuffield Department of Population Health, University of Oxford, Oxford, UK

**Keywords:** *Helicobacter pylori*, *H. pylori* antigens, CagA, non-cardia gastric cancer (NCGC), cardia gastric cancer (CGC), regional difference

## Abstract

**Background:**

*Helicobacter pylori* infection is a major cause of non-cardia gastric cancer (NCGC), but uncertainty remains about the associations between sero-positivity to different *H. pylori* antigens and risk of NCGC and cardia gastric cancer (CGC) in different populations.

**Methods:**

A case-cohort study in China included ∼500 each of incident NCGC and CGC cases and ∼2000 subcohort participants. Sero-positivity to 12 *H. pylori* antigens was measured in baseline plasma samples using a multiplex assay. Hazard ratios (HRs) of NCGC and CGC for each marker were estimated using Cox regression. These were further meta-analysed with studies using same assay.

**Results:**

In the subcohort, sero-positivity for 12 *H. pylori* antigens varied from 11.4% (HpaA) to 70.8% (CagA). Overall, 10 antigens showed significant associations with risk of NCGC (adjusted HRs: 1.33 to 4.15), and four antigens with CGC (HRs: 1.50 to 2.34). After simultaneous adjustment for other antigens, positive associations remained significant for NCGC (CagA, HP1564, HP0305) and CGC (CagA, HP1564, HyuA). Compared with CagA sero-positive only individuals, those who were positive for all three antigens had an adjusted HR of 5.59 (95% CI 4.68–6.66) for NCGC and 2.17 (95% CI 1.54–3.05) for CGC. In the meta-analysis of NCGC, the pooled relative risk for CagA was 2.96 (95% CI 2.58–3.41) [Europeans: 5.32 (95% CI 4.05–6.99); Asians: 2.41 (95% CI 2.05–2.83); *P_heterogeneity_*<0.0001]. Similar pronounced population differences were also evident for GroEL, HP1564, HcpC and HP0305. In meta-analyses of CGC, two antigens (CagA, HP1564) were significantly associated with a higher risk in Asians but not Europeans.

**Conclusions:**

Sero-positivity to several *H. pylori* antigens was significantly associated with an increased risk of NCGC and CGC, with varying effects between Asian and European populations.

Key Messages

- Sero-positivity to several *Helicobacter pylori* antigens was strongly associated with non-cardia gastric cancer (NCGC) and cardia gastric cancer (CGC), even among those infected with the known *H. pylori* virulence factor CagA.
- Sero-positivity to *H. pylori* antigens increases the risk of NCGC and CGC, with varying effects between Asian and European populations.
- Assessment of additional antibodies to *H. pylori* antigens may help identify individuals at high risk for gastric cancer.

## Introduction

Worldwide, gastric cancer is the fifth most frequently diagnosed cancer and the third leading cause of cancer death, responsible for >1 million new cases and 780 000 deaths in 2018.^1^ One of the most important and preventable causes of gastric cancer is *Helicobacter pylori* infection, which was responsible for an estimated 0.8 million new gastric cancer cases globally in 2018, with China alone accounting for more than 40% of *H. pylori* attributable cancers globally.^2^

Approximately 50% (over 3 billion) of the world population is estimated to be infected with *H. pylori*.^3^ However, the discrepancy between the large number of people infected and the minority of individuals who develop gastric cancer is thought to relate to a complex interplay of environmental risk factors, host susceptibility and differing virulence among *H. pylori* strains.^4^^,^^5^ This virulence can be partially explained by different *H. pylori* strains exhibiting variations in the presence or absence of virulence factors such as the cytotoxin-associated genes (*cag*) pathogenicity island, which encodes the oncogenic effector protein CagA, as well as allelic variation in the vacuolating cytotoxin A (VacA). There is evidence that different proteins of *H. pylori* induce different changes within the gastric mucosa, including invasion, survival, colonization and stimulation of further inflammation, all of which may contribute to the precancerous cascade leading to progression to gastric cancer.^5^ Moreover, in a recently reported case-cohort study using a sensitive immunoblot assay, there was a tendency for greater relative risks associated with larger numbers of more specific *H. pylori* antigens including CagA, VacA and UreaseA.^6^ Thus, detecting antibodies against specific antigens of *H. pylori* can help to identify individuals at high risk for gastric cancer.

Previous multiplex serological studies detecting antibody responses to specific *H. pylori* virulence factors have demonstrated association of *H. pylori* infection with non-cardia gastric cancer (NCGC), the reported relative risk estimates varying considerably for individual antigens.^7–13^ However, these studies tended to be case-control studies or prospective studies with relatively short follow-up time or with low case numbers. Moreover, these studies were mainly focused on NCGC and there remains substantial uncertainty about the role of different *H. pylori* infection antigens in the aetiology of cardia gastric cancer (CGC), which accounts for over one-sixth of gastric cancer cases globally.^14^

Using multiplex serology and a prospective case-cohort study design within the prospective China Kadoorie Biobank (CKB), we aimed to assess the associations between sero-positivity to different *H. pylori* antigens and risks of NCGC and CGC in Chinese adults. A further systematic review and meta-analysis were conducted by including seven other studies that used the same assay.

## Methods

### Study population

Details of the design, methods and study participants in the CKB have been previously described.^15^ Briefly, the baseline survey was conducted between 2004 and 2008 in 10 (five urban, five rural) geographically defined and diverse areas across China and enrolled 512 715 adults aged 30–79 years. Ethics approval was obtained from relevant international, national and local ethics committees. All participants provided written informed consent.

### Data collection

Extensive data were collected through a laptop-based questionnaire (e.g. sociodemographic, lifestyle and dietary factors and medical history) and physical measurements (e.g. blood pressure, adiposity), along with collection of a 10-ml blood sample for long-term storage.

### Follow-up for disease incident cases

Follow-up of CKB participants was done through linkage via unique personal ID numbers to established mortality and morbidity registries and to the nationwide health insurance system, which has almost universal coverage in study areas and records any episodes of hospitalization. Individuals were followed up until the first occurrence of the disease studied and were censored if they died of other causes, were lost to follow-up or reached the general censoring date, whichever occurred earlier.

By 1 January 2017, 44 037 (8.6%) of participants had died, 4781 (0.9%) were lost to follow-up and 27 903 developed cancer, including 3464 gastric cancers [International Classification of Diseases 10th Revision (ICD-10): C16]. For any reported cancer cases, systematic validation is being undertaken through retrieval and review of original medical records (including histopathological reports). Among the 1355 reported gastric cancer cases that have been adjudicated, 92% were confirmed, among which 85% were adenocarcinoma subtype.

### Design of case-cohort study

The design of our case-cohort study has been reported previously.^6^^,^^16^ In brief, incident gastric cancer cases were selected among those who were alive with no history of cancer 2 years after entering the study. Among all confirmed gastric cancer cases, we randomly selected 500 NCGC cases (out of 762 adjudicated cases) and all 437 CGC cases (ICD-10: C16.0) recorded. A subcohort of 2000 participants was sampled from the ‘modified baseline’ cohort, based on the following criteria: (i) alive and cancer-free for at least the first 2 years after study entry; and (ii) included in a random genotyped sample of the CKB cohort (∼80 000 participants) (Supplementary Figure S1, available as Supplementary data at *IJE* online).

### Multiplex serology

The multiplex serology panel used in our case-cohort study included a total of 12 *H. pylori* antigens (see details in Supplementary Table S1, available as Supplementary data at *IJE* online) and was adapted from the UK Biobank panel.^16^^,^^17^ The quantitative multiplex antibody detection approach combines the principle of a glutathione S-transferase (GST) capture enzyme-linked immunosorbent assay with fluorescent-bead technology.^18–20^ For this purpose, *H. pylori* antigens were bacterially expressed as GST-X-tag fusion proteins and loaded onto glutathione-casein covered beads. As these beads were spectrally distinguishable, it was possible to measure antibody levels to all antigens simultaneously for each of the study sera. The median fluorescence intensity (MFI) derived from at least 100 beads per antigen measured in a final serum dilution of 1:1000. Raw MFI values were corrected for antigen-specific background and patient-specific background reactivities. A sample was defined sero-positive for a specific antibody response against the corresponding antigen if the respective net MFI value exceeded the antigen-specific cut-off (Supplementary Figure S2, available as Supplementary data at *IJE* online). Antigen-specific cut-offs were applied as described previously,^21^ and were quality assured by the visual inflection point method.^22^ Overall *H. pylori* sero-positivity (*H. pylori*+) was defined as being sero-positive to any four or more of the 12 included *H. pylori* antigens.^16^

### Statistical analysis

Cumulative distribution functions were plotted to visually inspect the distributions of antibody levels (MFI) by study arm. Cox proportional hazards models were fitted using the Prentice pseudo-partial likelihood to estimate hazard ratios (HRs) for NCGC and CGC associated with seropositivity to each of the 12 *H. pylori* antigens, adjusting for age, sex and area (10 areas), with time in study used as the time scale.

To investigate the combined and independent effects of different antigens, further analyses were also done by the number of sero-positive *H. pylori* antigens (ranging from 0 to 12) and by simultaneous adjustment for each other in the models, resulting in associations for only three *H. pylori* antigens: HP1564, CagA and HP0305 for NCGC, and HP1564, CagA and HyuA for CGC). Because CagA is an established virulence marker for gastric cancer risk, we undertook an additional analysis of CagA with a few other antigens for both NCGC and CGC among individuals sero-positive to CagA, to examine their joint effects in those generally accepted high-risk populations. Collinearity between multiple risk factors was assessed using variance inflation factor (VIF), and value of VIF that exceed 10 was often regarded as indicating collinearity.

Analysis was done using R version 4.1.1.

### Systematic review and meta-analysis

This systematic review was undertaken according to the Preferred Reporting Items for Systematic Reviews and Meta-Analyses (PRISMA) guidelines.^23^ The protocol was registered in PROSPERO (CRD42022303678). Studies were identified by literature searches of relevant English language reports published prior to 1 January 2022 through PubMed databases, using search terms *Helicobacter pylori*, *H. pylori*, stomach neoplasms, gastric cancer and multiplex serology.

Studies were eligible for inclusion if: (i) they were cohort or case-control studies that reported the associations of different *H. pylori* antigens with risk of gastric cancer; (ii) antibody levels against multiple *H. pylori* antigens were detected by multiplex serology; and (iii) the study was conducted in an adult population. Studies without antigen-specific risk estimates data for retrieval and duplicate publications were excluded.

Data were extracted from each report using a predefined review form, covering ethnicity of participants, country of study, types of study design, age distributions, follow-up time, model information, antigen-specific sero-prevalence and relative risk (RR 95% CIs), where applicable.

Antigen-specific results of individual studies were meta-analysed with the pooled RRs estimated using an inverse-variance weighted fixed-effects model. Meta-regression analyses were conducted to assess the relationship between sero-prevalence and RR across studies. Heterogeneity was assessed using the *I*² statistic and Cochran’s *Q* test. Publication bias was assessed visually by inspecting the funnel plots for asymmetry and tested with Egger’s test.^24^ Furthermore, a sensitivity analysis was also conducted to test the influence of one study from West Asia (Iran) in Asian subgroups.

## Results

### Characteristics of the CKB population

Among the 935 prospectively ascertained cases of gastric cancer and the 1998 subcohort participants (12 samples were excluded due to technical errors) in CKB, the mean age at study entry was 59.4 years and the average follow-up time from blood collection to diagnosis was 8.1 years among cases. Compared with the subcohort participants, NCGC cases had a higher proportion of women and were more likely to be urban residents, to have higher socioeconomic status, to be regular smokers or alcohol drinkers (among men), to consume meat, fresh fruit and preserved vegetables and to report good health at baseline (Table 1). For CGC cases, however, the converse appeared to be true except for preserved vegetables, for which both case arms showed similar patterns compared with subcohort participants.

**Table 1:** Baseline characteristics of cases and subcohort participants in the China Kadoorie Biobank

Characteristics^a^	NCGC	CGC	Subcohort
	(*n* = 499)	(*n* = 436)	(*n* = 1998)
Demographic factors			
Age (years), mean (SD)	58.9 (9.5)	61.1 (8.6)	59.1 (9.9)
Women, %	35.2	25.5	30.6
Urban residents, %	71.8	36.6	50.4
≥6 years of education, %	45.1	32.5	43.4
Household income (>20000 CNY/year), %	46.6	30.3	42.0
Lifestyle factors			
Current regular smoking, %			
Men	70.3	57.1	58.2
Women	2.9	0	3.9
Current regular alcohol drinking, %			
Men	41.0	27.3	32.0
Women	1.1	0	4.6
Physical activity (MET h/day), mean (SD)	19.4 (14.8)	18.2 (15.5)	17.9 (14.0)
Medical history and health status, %			
Poor self-rated health	8.5	12.2	11.0
Diabetes (self-reported or screen detected)	6.8	5.7	8.8
Prior peptic ulcer	6.6	5.3	5.4
Prior cirrhosis/chronic hepatitis	0.6	0.7	1.2
Prior CHD/stroke/TIA	5.6	7.3	10.4
Prior emphysema/bronchitis	2.4	4.1	4.2
Anthropometry and blood pressure, mean (SD)			
BMI (kg/m^2^)	23.6 (3.4)	24.0 (3.5)	23.7 (3.5)
Waist circumference (cm)	81.2 (10.0)	82.3 (10.2)	81.4 (10.3)
SBP (mmHg)	135 (23)	138 (23)	136 (22)
Daily dietary consumption , %			
Rice	67.5	41.9	71.6
Wheat	44.3	62.2	37.6
Meat	34.9	16.0	30.0
Poultry	1.2	0.2	0.4
Fresh vegetables	95.0	97.3	96.2
Soybean	4.8	3.2	3.6
Preserved vegetables	32.1	24.3	17.2
Fresh fruit	25.1	7.6	19.4
Spicy food	17.2	9.4	24.4

BMI, body mass index; CGC, cardia gastric cancer; CHD, coronary heart disease; CNY, Chinese Yuan; MET-h, metabolic equivalent of task-hours; NCGC, non-cardia gastric cancer; SBP, systolic blood pressure; SD, standard deviation; TIA, transient ischaemic attack.

a Adjusted, where appropriate, for age, sex and areas.

### Associations of *H. pylori* antigens with risks of gastric cancers in CKB

**Figure 1:**
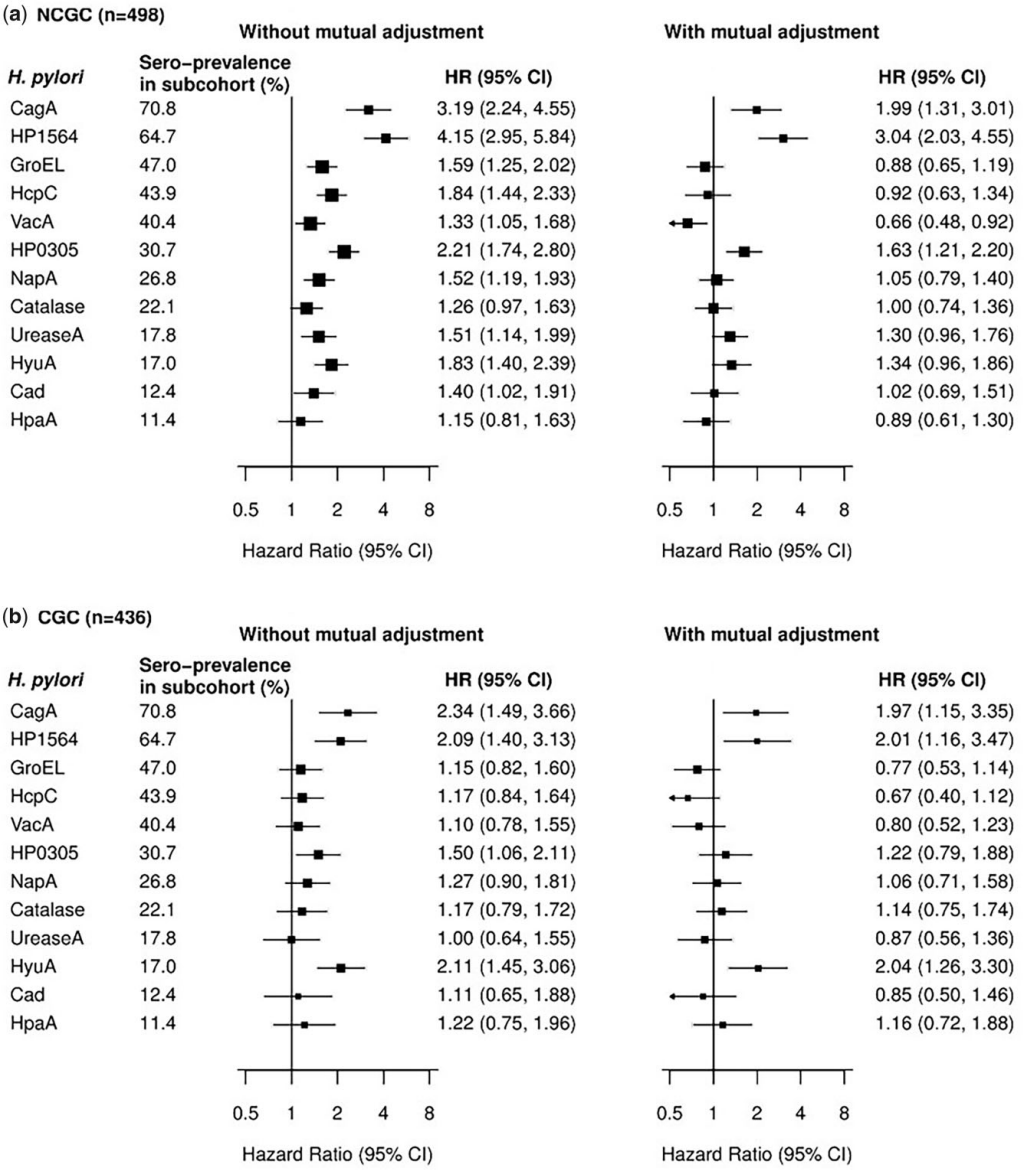
Associations of antibodies to *Helicobacter pylori* antigens with risks of a) non-cardia gastric cancer (NCGC) and b) cardia gastric cancer (CGC), with and without mutual adjustment for different antigens. Antigens are ordered by sero-prevalence in the subcohort, from high to low. The adjusted hazard ratio (HR) for each antibody to *H. pylori* antigen was assessed using Cox proportional hazards models, using the Prentice pseudo-partial likelihood. Models were adjusted for age, sex and area (10 areas), with or without mutual adjustment

Of the 12 *H. pylori* antigens investigated in CKB, the sero-prevalence of individual antigens in the subcohort ranged from 11.4% (HpaA) to 70.8% (CagA). Compared with participants who were sero-negative to each relevant antigen, sero-positivity to each of 10 individual *H. pylori* antigens (HP1564, CagA, HP0305, HcpC, HyuA, GroEL, UreaseA, NapA, Cad, VacA) was significantly associated with a higher risk of NCGC, with HRs ranging from 1.33 (95% CI 1.05–1.68) for VacA to 4.15 (95% CI 2.95–5.84) for HP1564 (Figure 1). For CGC, sero-positivity to each of four antigens (CagA, HyuA, HP1564, HP0305) was significantly associated with a higher risk, with HRs ranging from 1.50 (95% CI 1.06–2.11) for HP0305 to 2.34 (95% CI 1.49–3.66) for CagA.

The mean number of sero-positive antigens was 4.0 (SD 2.9) in the subcohort, 5.7 (2.6) in NCGC and 5.3 (2.7) in CGC (Supplementary Figure S3, available as Supplementary data at *IJE* online). The HRs appeared to increase significantly with the numbers of sero-positive antigens but levelled off beyond four sero-positive antigens from any of the 12 antigens (Supplementary Figure S4, available as Supplementary data at *IJE* online).

No major collinearity was observed regarding the sero-status of these 12 *H. pylori* antigens (VIF≤2.4). After simultaneous adjustment for other antigens in the model, positive associations persisted for NCGC (CagA, HP1564, HP0305) and CGC (CagA, HP1564, HyuA) (Figure 1). When we restricted analyses to individuals who were sero-positive to CagA, sero-positivity to either HP1564 or HP0305 had an over 3-fold higher risk of NCGC (3.60, 95% CI 2.96–4.36) rising to over 5-fold higher risk (5.59, 95% CI 4.68–6.66) when both antigens were sero-positive (Table 2). Similar, albeit less extreme, associations were observed for CGC, with those who were positive for both HP1564 and HyuA having an HR of 2.17 (95% CI 1.54–3.05).

**Table 2:** Adjusted hazard ratios (HRs) for a) non-cardia gastric cancer (NCGC) and b) cardia gastric cancer (CGC) by sero-status of selected key antigens among 1854 individuals who were sero-positive to the *Helicobacter pylori* antigen CagA

	Proportion in subcohort, %	HR (95%CI)	χ^2^ (*P* for trend)
a) NCGC			
HP1564- and HP0305-	14	1.00 (0.60–1.67)	
HP1564+ or HP0305+	42	3.60 (2.96–4.36)	
HP1564+ and HP0305+	44	5.59 (4.68–6.66)	36.4 (<0.0001)
b) CGC			
HP1564- and HyuA-	16	1.00 (0.61–1.65)	
HP1564+ or HyuA+	61	1.13 (0.89–1.45)	
HP1564+ and HyuA+	23	2.17 (1.54–3.05)	9.1 (0.0026)

### Systematic review and meta-analysis of CKB with published studies

Our systematic review of *H. pylori* antigens and gastric cancer identified 29 potentially eligible studies, of which seven met the inclusion criteria (Supplementary Figure S5, available as Supplementary data at *IJE* online).^7–13^ To note, in the Linxian General Population Nutrition Intervention Trial (NIT),^10^ there were two analytical datasets (blood samples were drawn in 1999/2000 or 1985) with different study designs and without overlapping cases or controls. Only the 1999/2000 set was included in the meta-analysis, as the 1985 set was already included in another study.^12^ Of these seven studies that reported associations of *H. pylori* antigens with risks of NCGC, four were conducted in Asia (2055 NCGC cases) and three in Europe (520 NCGC cases). These included four case-control studies, one nested case-control study, one case-cohort study and one pooled analysis of nested case-control studies in East Asia. Selected characteristics of these seven studies are shown in Supplementary Table S3 (available as Supplementary data at *IJE* online). The NCGC case number of individual studies varied from 100 to 1608, mean age at entry was 64 (range 57–68) years. Three prospective studies reported average follow-up time of 3.6–7.0 years from blood collection to diagnosis. However, only three of these seven studies included CGC cases (total 251 cases).

**Figure 2:**
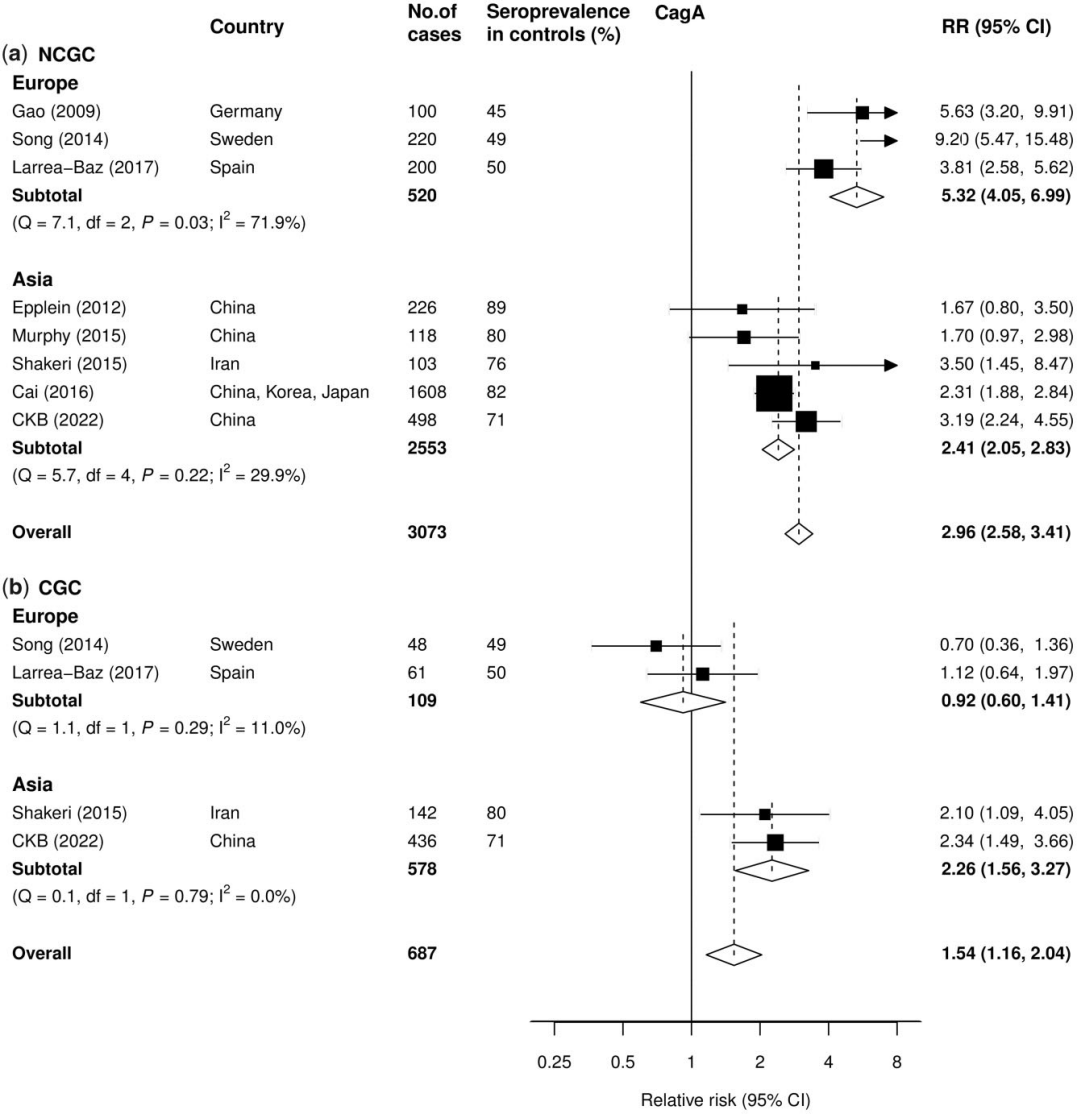
Adjusted relative risks of sero-positivity to the *Helicobacter pylori* antigen CagA for a) non-cardia gastric cancer (NCGC) and b) cardia gastric cancer (CGC) in a meta-analysis of the China Kadoorie Biobank and seven published studies, by region. Boxes represent the relative risks (RRs) associated with sero-positivity of antibody to *H. pylori* CagA for individual studies, with the size of the box inversely proportional to the variance of the logRR. Diamonds represent summary relative risks for each region and overall. Within categories RRs are ordered according to their year of publication. Estimates and 95% CI of the summary RRs are in bold.

A meta-analysis comprising the CKB and the additional seven studies showed that sero-positivity to CagA was associated with a pooled RR of 2.96 (95% CI 2.58–3.41) for NCGC (3073 cases; Figure 2) with a significant heterogeneity in RRs between individual studies (I^2^* *=* *82.4%, *P *<* *0.001). Moreover, the RRs for NCGC were higher in European (5.32, 95% CI 4.05–6.99) than in Asian (2.41, 95% CI 2.05–2.83) studies. There was no evidence of heterogeneity between the five Asian studies (I^2^=29.9%, *P *=* *0.22), but there was heterogeneity between the three European studies (I^2^* *=* *71.9%, *P *=* *0.03).

In contrast, only sero-positivity to CagA was significantly associated with a high risk of CGC (1.54, 95% CI 1.16–2.04), with the positive association confined mainly to Asian (2.09, 95% CI 1.48–2.94) but not European (0.92, 95% CI 0.60–1.41) studies (Figure 2). There was no heterogeneity between studies conducted within Europe or Asia, respectively.

Among the eight studies, sero-prevalence of CagA was 45–50% in Europe and 71–89% in Asia. There was large heterogeneity in RR estimates across different studies by CagA sero-prevalence in controls or subcohorts, for both NCGC (regression slope = -0.03, 95% CI -0.04 to -0.02, *P *<* *0.0001) and CGC (regression slope = 0.03, 95% CI 0.01–0.05, *P *=* *0.003; Supplementary Figure S6, available as Supplementary data at *IJE* online). There was no evidence of publication bias in studies of NCGC (Egger’s test *P *=* *0.41), or CGC (Egger’s test *P *=* *0.38) (Supplementary Figure S7, available as Supplementary data at *IJE* online).

**Figure 3:**
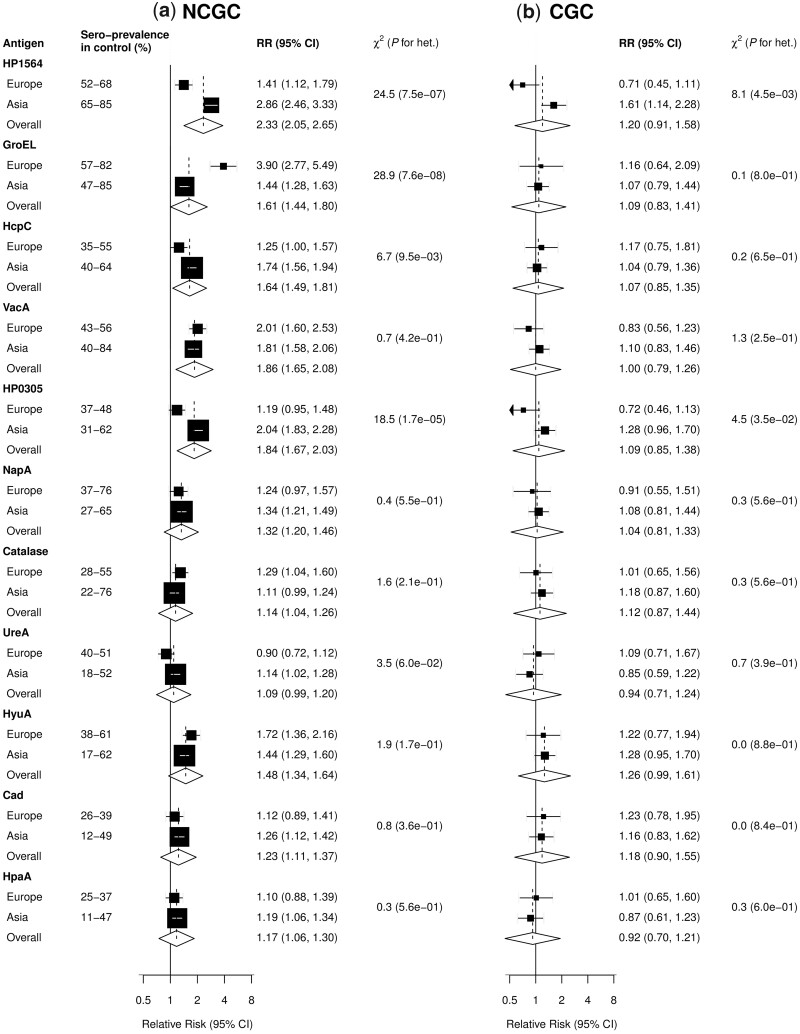
Adjusted relative risks of sero-positivity to different *Helicobacter pylori* antigens for a) non-cardia gastric cancer (NCGC) and b) cardia gastric cancer (CGC) in a meta-analysis of the China Kadoorie Biobank and seven published studies, by region. Boxes represent the summary relative risks (RRs) associated with sero-positivity of antibody to *H. pylori* antigens for each region, with the size of the box inversely proportional to the variance of the logRR. Diamonds represent summary relative risks overall. For each antigen, there were three studies in Europe (520 cases) and five studies in Asia (2553 cases) for NCGC, two studies in Europe (109 cases) and two studies in Asia (578 cases) for CGC

In addition to CagA, sero-positivity to each of the other 10 antigens (HP1564, GroEL, HcpC, VacA, HyuA, HP0305, NapA, Cad, HpaA and Catalase) was significantly associated with risk of NCGC, with pooled RRs ranging from 1.14 (95% CI 1.04–1.26) for Catalase to 2.33 (95% CI 2.05–2.65) for HP1564 (Figure 3). The pooled RRs were significantly higher in Europe than Asia studies for GroEL [3.90 (95% CI 2.77–5.49) vs 1.44 (95% CI 1.28–1.63)], whereas the converse was true for HP1564 [1.41 (95% CI 1.12–1.79) vs 2.86 (95% CI 2.46–3.33)], HcpC [1.25 (95% CI 1.00–1.57) vs 1.74 (95% CI 1.56–1.94)] and HP0305 [1.19 (95% CI 0.95–1.48) vs 2.04 (95% CI 1.83–2.28)].

For CGC, HP1564 was also significantly associated with increased risk in Asian but not European studies [1.61 (95% CI 1.14–2.28) vs 0.71 (95% CI 0.45–1.11)], *P* for heterogeneity = 0.0045; Figure 3). In meta-analyses, none of the other examined antigens was found to be significantly associated with risk of CGC, either overall or in Asian or European populations separately (Figure 3; Supplementary Figures S8–S18, available as Supplementary data at *IJE* online). In sensitivity analyses after excluding one study from Iran, there was no material change in the observed associations (Supplementary Figure S19, available as Supplementary data at *IJE* online).

## Discussion

This large prospective case-cohort study suggested that antibody responses to specific *H. pylori* antigens were strong risk factors not only for NCGC but also for CGC in Chinese adults. In addition to the well-established *H. pylori* virulence factor CagA, antibody response to HP1564 (referred to as OMP in previous studies) or HP0305 was associated with an increased risk of NCGC even among those who were CagA sero-positive. In a meta-analysis of CKB and seven other studies using the same assay, associations between sero-positivity to *H. pylori* antigens and NCGC were more profound in Europeans for CagA and GroEL, and in Asians for HP1564, HcpC and HP0305. For CGC, sero-positivity to two antigens (CagA and HP1564) were found to be significantly associated with increased risk among Asians but not Europeans.

In the CKB, sero-positivity to each of 10 (HP1564, CagA, HP0305, HyuA, HcpC, GroEL, UreA, NapA, Cad, VacA) out of the 12 *H. pylori* antigens examined was significantly associated with a higher risk of NCGC. Similar findings were also reported in previous studies from Asian or European populations.^7–13^ Moreover, even among the already established high-risk group of individuals who were sero-positive to the known virulence factor CagA, dual HP1564/HP0305 sero-positivity remained strongly associated with an over 5-fold increased risk of NCGC. A similar but less striking difference (over 3-fold increase) was also shown in a pooled analysis involving 1608 NCGC cases and 1958 controls in the high-risk East Asian population.^12^ These consistent associations with HP1564 and HP0305 may be indicative of the relatively stronger antibody reactions to these two antigens during chronic *H. pylori* infection.

To date, seven studies have assessed the associations of different *H. pylori* antigens (*n* = 15–17) with risk of gastric cancer using multiplex serology.^7–13^ However, most of them were case-control studies with samples collected after cancer diagnosis, or prospective studies with short follow-up. Moreover, the majority of these studies focused on NCGC only. A recent meta-analysis of seven studies identified 5 *H. pylori* virulence factors (CagA, HP0305, HyuA, HP1564 and VacA) that were significantly associated with risk of NCGC but not with CGC.^25^ Among the included studies in the recent meta-analysis, the study by Murphy *et al.* included two analytical sets of cases and controls (1985 analytical set and 1999 analytical set) with no overlap and thus were considered as two independent studies.^10^ However, the 1985 analytical set had also been included in a previous pooled analysis by Cai *et al*.,^12^ resulting in duplication in this meta-analysis. Our meta-analyses, with the exclusion of the above duplicated study and additionally inclusion of the CKB findings, has identified 11 antigens (including five antigens aforementioned) that were associated with NCGC in both European and Asian populations, and one antigen for CGC, primarily in Asian populations.

*H. pylori* CagA was the first identified bacterial protein involved in human cancer.^26^ This oncoprotein is delivered into gastric epithelial cells via bacterial type IV secretion and provokes premature cell senescence and/or programmed cell death (apoptosis). Our findings reinforce the notion that CagA-positive *H. pylori* is highly prevalent in Asian compared with European countries. Our risk estimate for NCGC associated with CagA was consistent with studies in other Asian populations, and meta-analysis by regions indicated that the effect size of CagA was more than twice as strong in European as in Asian populations. Moreover, regional heterogeneity in risk estimates were also observed between NCGC and *H. pylori* antigens of GroEL, HP1564, HcpC and HP0305. The reasons for such large difference in risk estimates are not fully understood, but may reflect differences in the sero-prevalence of *H. pylori* antigens which were generally higher in Asian populations than in European populations (CagA: 71–89% in Asians vs 45–50% in Europeans; HP1564: 65–85% in Asian vs 52–68% in Europeans), indicating potentially a relatively more important role of other factors in the aetiology of gastric cancer in Chinese populations. Another potential explanation is the structural diversity in the C-terminal half of CagA, where a Glu-Pro-Ile-Tyr-Ala (EPIYA) host-interacting motif is repeated.^26^ The Western version of CagA carries the EPIYA segment types A, B and C, whereas the East Asian CagA carries types A, B and D, and thus this variability of polymorphic EPIYA may account for differences in the effect size of the pathogenicity of *H. pylori* strains among different populations.^26^ High heterogeneity between the three European studies was observed with a stronger effect estimate reported by Song *et al.* (OR = 9.20, 95% CI 6.23–13.59) compared with the other two studies (3.81, 95% CI 2.58–5.62 and 5.63, 95% CI 3.20–9.91).^7^^,^^9^^,^^13^

Previously studies reported null associations of the *H. pylori* antigens examined and risk of CGC in European (*n* = 2) or Asian (*n *= 1) populations. Using the same multiplex serology, CKB found that sero-positivity to each of four antigens (CagA, HyuA, HP1564, HP0305) was associated with increased risk of CGC. This discrepancy may be partly due to the small number of cases included in the previous studies (CKB included about twice as many cases as in all the three studies combined) and collection of blood samples near cancer diagnosis (as opposed to at least 2 years before cancer diagnosis in CKB). Moreover, it may also partly be attributed to the fact that there are two distinct aetiologies of CGC.^1^ One type of CGC resembles oesophageal adenocarcinoma and is associated with gastrooesophageal reflux mainly appearing in Western populations. Another type resembles NCGC, being a consequence of atrophic gastritis due to *H. pylori* infection and concentrating in East Asian populations.

The strengths of our study included prospective design with exclusion of the first 2 years after blood collection to limit reverse causation, and well-characterised and large number of cases of different subtypes of gastric cancer. The use of a multiplex serology assay enabled a comprehensive assessment of multiple *H. pylori* antigens in gastric cancer risk individually and collectively. Moreover, we also undertook a systematic review and meta-analysis of the studies that used the same assay method. However, the study also has limitations. First, studies included were confined to only Asian and European populations, which may limit the generalizability of the results to other populations. Second, residual confounding may still exist.

## Conclusion

In summary, our study provides new evidence about the role of sero-positivity to multiple *H. pylori* antigens in the association with both NCGC and CGC in China where *H. pylori* infection is widespread. Antigens of HP1564 and HP0305 are strongly associated with risk of NCGC even among those individuals infected and sero-positive to the known gastric cancer virulence factor CagA. Different patterns of association between *H. pylori* antigens and NCGC or CGC were identified between Asian and European populations. As *H. pylori* multiplex serology is a non-invasive test with high throughput capacity, it has potential, in combination with other predictive factors, for translation into targeted prevention strategies to reduce the morbidity and mortality of one of the world’s most deadly cancers.

## Ethics approval

Ethics approval was obtained from Oxford University, the China National CDC and also from institutional research boards at the local CDCs in the 10 areas. All participants provided written informed consent.

## Supplementary Material

dyad007_Supplementary_Data

## Data Availability

Anonymized baseline, resurvey and cause-specific mortality and morbidity data are available for access through a formal application in the CKB website [http://www.ckbiobank.org]. The application will then be reviewed by a data access committee. Further details about access policy and procedures can be found online at [http://www.ckbiobank.org].

## Open Access statement reminder

This research was funded in whole, or in part, by the Wellcome Trust [212946/Z/18/Z, 202922/Z/16/Z, 104085/Z/14/Z, 088158/Z/09/Z]. For the purpose of Open Access, the author has applied a CC-BY public copyright licence to any Author Accepted Manuscript version arising from this submission.

## References

[1] Bray F , Ferlay J, Soerjomataram I et al Global cancer statistics 2018: GLOBOCAN estimates of incidence and mortality worldwide for 36 cancers in 185 countries. CA Cancer J Clin 2018;68:394–424. 30207593 10.3322/caac.21492

[2] de Martel C , Georges D, Bray F et al Global burden of cancer attributable to infections in 2018: a worldwide incidence analysis. Lancet Glob Health 2020;8:e180–90. 31862245 10.1016/S2214-109X(19)30488-7

[3] Franceschi S , Herrero R. Infections. In: Steward BW, Wild CP, eds. World Cancer Report 2014. Lyon, France: International Agency for Research on Cancer, 2014, pp.105–107.

[4] Correa P , Houghton J. Carcinogenesis of Helicobacter pylori. Gastroenterology 2007;133:659–72. 17681184 10.1053/j.gastro.2007.06.026

[5] Wroblewski LE , Peek RM Jr, Wilson KT. Helicobacter pylori and gastric cancer: factors that modulate disease risk. Clin Microbiol Rev 2010;23:713–39. 20930071 10.1128/CMR.00011-10 PMC2952980

[6] Yang L , Kartsonaki C, Yao P et al The relative and attributable risks of cardia and non-cardia gastric cancer associated with *Helicobacter pylori* infection in China: a case-cohort study. Lancet Public Health 2021;6:e888–96. 34838195 10.1016/S2468-2667(21)00164-X PMC8646857

[7] Gao L , Michel A, Weck MN et al Helicobacter pylori infection and gastric cancer risk: evaluation of 15 H. pylori proteins determined by novel multiplex serology. Cancer Res 2009;69:6164–70. 19602590 10.1158/0008-5472.CAN-09-0596

[8] Epplein M , Zheng W, Xiang YB et al Prospective study of Helicobacter pylori biomarkers for gastric cancer risk among Chinese men. Cancer Epidemiol Biomarkers Prev 2012;21:2185–92. 23035179 10.1158/1055-9965.EPI-12-0792-T PMC3518572

[9] Song H , Michel A, Nyrén O et al A CagA‐independent cluster of antigens related to the risk of noncardia gastric cancer: associations between Helicobacter pylori antibodies and gastric adenocarcinoma explored by multiplex serology. Int J Cancer 2014;134:2942–50. 24259284 10.1002/ijc.28621

[10] Murphy G , Freedman ND, Michel A et al Prospective study of Helicobacter pylori antigens and gastric noncardia cancer risk in the nutrition intervention trial cohort. Int J Cancer 2015;137:1938–46. 25845708 10.1002/ijc.29543 PMC4529753

[11] Shakeri R , Malekzadeh R, Nasrollahzadeh D et al Multiplex H. pylori serology and risk of gastric cardia and noncardia adenocarcinomas. Cancer Res 2015;75:4876–83. 26383162 10.1158/0008-5472.CAN-15-0556 PMC4792189

[12] Cai H , Ye F, Michel A et al Helicobacter pylori blood biomarker for gastric cancer risk in East Asia. Int J Epidemiol 2016;45:774–81. 27170766 10.1093/ije/dyw078 PMC5841796

[13] Fernández de Larrea-Baz N , Pérez-Gómez B, Michel A et al Helicobacter pylori serological biomarkers of gastric cancer risk in the MCC-Spain case-control study. Cancer Epidemiol 2017;50:76–84. 28888185 10.1016/j.canep.2017.08.002

[14] Arnold M , Ferlay J, van Berge Henegouwen MI et al Global burden of oesophageal and gastric cancer by histology and subsite in 2018. Gut 2020;69:1564–71. 32606208 10.1136/gutjnl-2020-321600

[15] Chen Z , Chen J, Collins R et al; China Kadoorie Biobank (CKB) Collaborative Group. China Kadoorie Biobank of 0.5 million people: survey methods, baseline characteristics and long-term follow-up. Int J Epidemiol 2011;40:1652–66. 22158673 10.1093/ije/dyr120 PMC3235021

[16] Yao P , Millwood I, Kartsonaki C et al Sero-prevalence of 19 infectious pathogens and associated factors among middle-aged and elderly Chinese adults: a cross-sectional study. BMJ Open 2022;12:e058353. 10.1136/bmjopen-2021-058353 PMC9086621 35534062

[17] Mentzer AJ , Brenner N, Allen N et al; UKB Infection Advisory Board. Identification of host-pathogen-disease relationships using a scalable multiplex serology platform in UK Biobank. Nat Commun 2022;13:1818. 35383168 10.1038/s41467-022-29307-3 PMC8983701

[18] Waterboer T , Sehr P, Michael KM et al Multiplex human papillomavirus serology based on in situ–purified glutathione S-transferase fusion proteins. Clin Chem 2005;51:1845–53. 16099939 10.1373/clinchem.2005.052381

[19] Brenner N , Mentzer AJ, Butt J et al Validation of multiplex serology detecting human herpesviruses 1-5. PLoS One 2018;13:e0209379. 30589867 10.1371/journal.pone.0209379 PMC6307738

[20] Brenner N , Mentzer AJ, Butt J et al Validation of multiplex serology for human hepatitis viruses B and C, human T-lymphotropic virus 1 and Toxoplasma gondii. PLoS One 2019;14:e0210407. 30615688 10.1371/journal.pone.0210407 PMC6322760

[21] Michel A , Waterboer T, Kist M et al Helicobacter pylori multiplex serology. Helicobacter 2009;14:525–35. 19889070 10.1111/j.1523-5378.2009.00723.x

[22] Migchelsen SJ , Martin DL, Southisombath K et al Defining seropositivity thresholds for use in trachoma elimination studies. PLoS Negl Trop Dis 2017;11:e0005230. 28099433 10.1371/journal.pntd.0005230 PMC5242428

[23] Page MJ , McKenzie JE, Bossuyt PM et al The PRISMA 2020 statement: an updated guideline for reporting systematic reviews. BMJ 2021;372:n71. 33782057 10.1136/bmj.n71 PMC8005924

[24] Egger M , Smith G, Schneider M et al Bias in meta-analysis detected by a simple, graphical test. BMJ 1997;315:629–34. 9310563 10.1136/bmj.315.7109.629 PMC2127453

[25] El Hafa F , Wang T, Ndifor VM et al Association between Helicobacter pylori antibodies determined by multiplex serology and gastric cancer risk: A meta-analysis. Helicobacter 2022;27:e12881. 35212073 10.1111/hel.12881

[26] Hatakeyama M. Structure and function of Helicobacter pylori CagA, the first-identified bacterial protein involved in human cancer. Proc Jpn Acad Ser B Phys Biol Sci 2017;93:196–219. 10.2183/pjab.93.013 PMC5489429 28413197

