# Supplementary material for: Helicobacter pylori multiplex serology and risk of non-cardia and cardia gastric cancer: a case-cohort study and meta-analysis
Source: Int J Epidemiol. 2023 Mar 13;52(4):1197–208. doi: 10.1093/ije/dyad007 (PMC10396410; doi:10.1093/ije/dyad007)

| Table S1. List of *Helicobacter pylori* Multiplex Serology Antigens and cut-offs used for defining sero-positivity | | | |  |
| --- | --- | --- | --- | --- |
| **Antigen** | **Full name, function** | **Cut-off (MFI)** | **Criteria for overall sero-positivity** | |
| HyuA-C | hydantoin utilization protein A | 280 | ≥4 positives out of 12 | |
| GroEL | chaperonin GroEL | 500 |  |  |
| UreA | urease alpha subunit | 300 |  |  |
| CagA-N/C | cytotoxin-associated antigen A, pathogenesis | 1500 |  |  |
| Catalase |  | 300 |  |  |
| HP1564 | hypothetical protein | 150 |  |  |
| VacA-N/C | vacuolating cytotoxin, pathogenesis | 385 |  |  |
| NapA | neutrophil-activating protein | 120 |  |  |
| HP0305 | hypothetical protein | 130 |  |  |
| HpaA | neuraminyllactose-binding hemagglutinin homolog | 200 |  |  |
| Cad | cinnamyl alcohol dehydrogenase | 100 |  |  |
| HcpC | conserved hypothetical secreted protein | 150 |  |  |

CagA and VacA were each split into an N-terminal and C-terminal part. Respective readouts were summed up to obtain an overall seroresponse

| Table S2. Search strategy used for identifying relevant studies in meta-analyses | | |
| --- | --- | --- |
| **Meta-analysis** | **Database** | **Search strategy** |
| Studies | PubMed | #1 “*Helicobacter pylori*”[Title/Abstract] OR “*H. pylori*”[Title/Abstract]  #2 “gastric cancer”[Title/Abstract] OR “stomach neoplasms” [Title/Abstract]  #3 “multiplex serology”[Title/Abstract]  #3 #1 and #2 and #3  Filters: Humans; English |

| Table S3. Characteristics of studies included in the meta-analysis of *H. pylori* antigens and risk of gastric cancer | | | | | | | |  |
| --- | --- | --- | --- | --- | --- | --- | --- | --- |
| Study | Author (year) | Country | Dates of enrolment | Case | Prospective study design | Mean age at entry (years) | No. of antigens | |
| 1 | Gao (2009) | Germany | 1996-2003 | 100 NCGC | no | 65 | 15 | |
| 2 | Epplein (2012) | China (SMHS) | 2002-2006 | 226 NCGC | yes | 62 | 15 | |
| 3 | Song (2014) | Sweden | 1989-1995 | 220 NCGC 48 CGC | no | 68 | 17 | |
| 4 | Murphy (2015) | China (NIT) | 1999 | 118 NCGC | yes | 64 | 15 | |
| 5 | Shakeri (2015) | Iran | 2004-2011 | 103 NCGC 142 CGC | no | 65 | 15 | |
| 6 | Cai (2016) | China, Korea, Japan | 1985-2011 | 1608 NCGC | yes | 57 | 15 | |
| 7 | Larrea-Baz (2017) | Spain | 2008-2013 | 200 NCGC 61 CGC | no | 66 | 16 | |
| 8 | CKB (2022) | China | 2004-2008 | 498 NCGC 436 CGC | yes | 55 | 12 | |

### Figure S1. Flow diagram of study design and participant selection in CKB

*Selection for the subcohort used simple random sampling; †Individuals may be included in more than one study arm; CKB: China Kadoorie Biobank; CGC: cardia gastric cancer; NCGC: non-cardia gastric cancer.

**Study arm 2^†^**

NCGC cases

n=500

**Study arm 1^†^**

CGC cases

n=437

**Study arm 3^†^**

Subcohort

n=2000

Not selected (random)

n=262

Adjudicated

n=14,316

Not adjudicated

n=5,676

EAC cases

n=27

NCGC cases

n=762

CGC cases

n=136

CGC cases

n=301

Other cancer types

n=5,284

Confirmed cancer cases

n=14,167

Not confirmed as cancer

n=149

Other cancer types

n=13,333

Not selected* n=63,684

All CKB participants

n=512,715

Eligible CKB participants

n=452,957

Cancer history at baseline

n=2,411

Cancer event or died in first 2 years follow-up

n=6,150

Genotyped participants

n=65,684

Reported cancer cases

n=19,992

Not genotyped, or selection for genotyping not random

n=387,273

Plasma sample not available or previously used in other studies

n=51,197

### Figure S2. Cumulative proportions of study participants according to levels of antibodies to specific *H. pylori* antigens, by study arm

Cumulative distribution function were plotted to visually inspect the distributions of antibody levels (MFI) by case arm. For each antigen, orange line represents cut-off value.


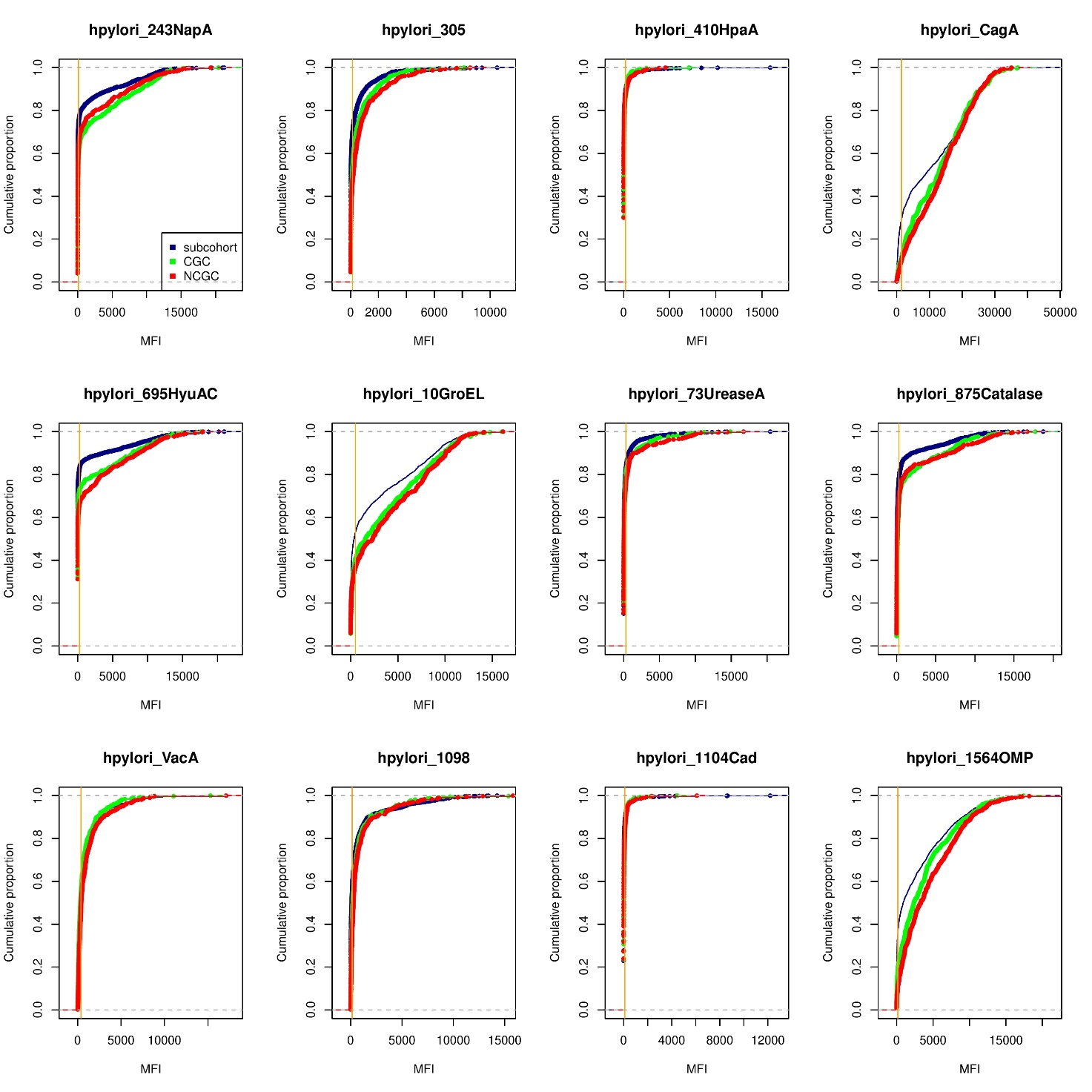


### Figure S3. Number of sero-positivities of H. pylori antigens in a) subcohort, b) non-cardia and c) cardia gastric cancer

The dashed lines represent the mean numbers of seropositive *H. pylori* antibodies among the study participants, which is 4.0 (SD 2.9) in subcohort, 5.7 (2.6) in NCGC, 5.3 (2.7) in CGC.


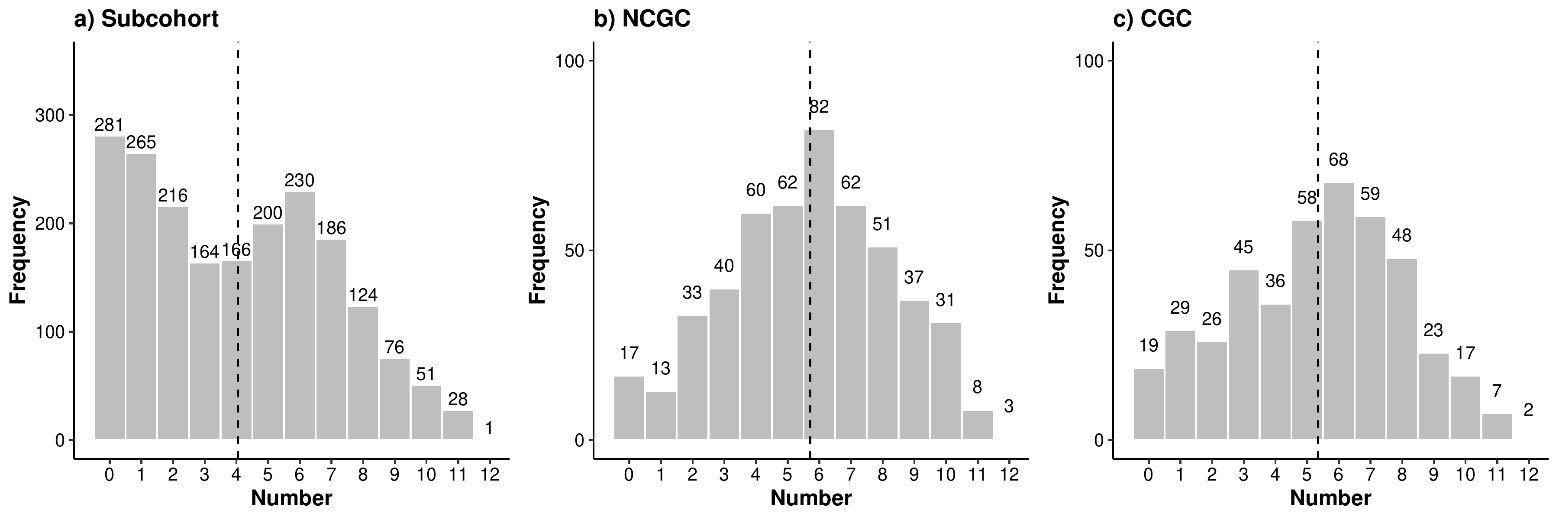


### Figure S4. Adjusted HRs for a) non-cardia and b) cardia gastric cancer associated with sero-positivities of different numbers of *H* *pylori* antigens

Adjusted HR for cumulative number of positive antigens were assessed using Cox proportional hazards models, using the Prentice pseudo-partial likelihood. Models were adjusted for age, sex and area (10 areas).


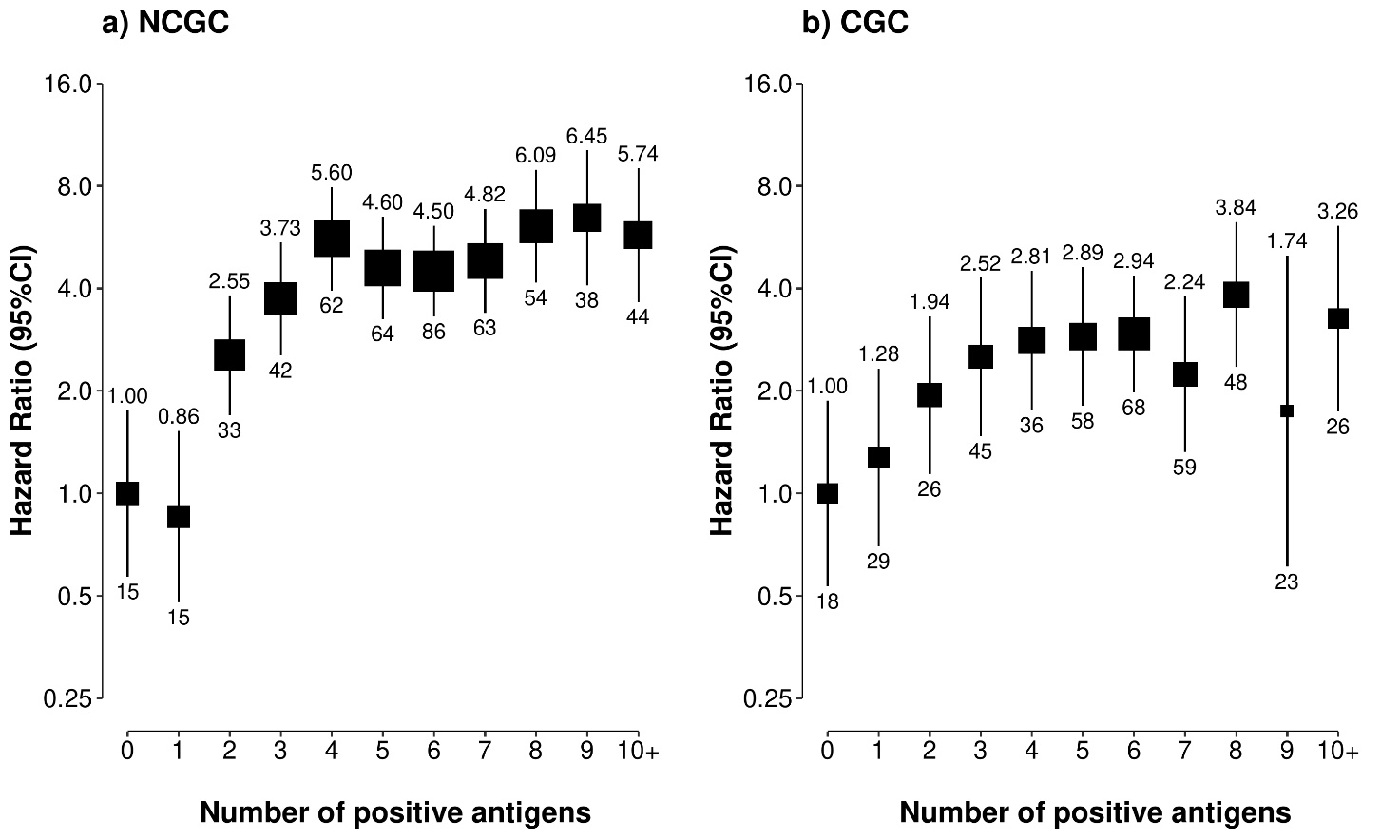


### Figure S5. Flow chart of literature search and review process for published studies investigating association between *H. pylori* antigens and risk of gastric cancer

###
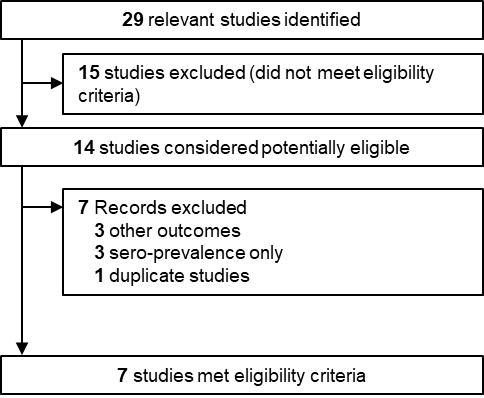


### Figure S6. Relative Risks (95% CI) for a) non-cardia GC and b) cardia GC by levels of sero-prevalence of CagA in controls or subcohorts of 8 studies included in meta-analyses

Solid points represent European studies and hollow ones represent studies in Asia. Different point symbols indicate the adjusted Relative Risk (RR) in individual studies, and the size of symbols is inversely proportional to the variance of the RR. The dashed line depicts the estimated linear relationship with 95% CI (grey area) between RR of gastric cancer associated with CagA sero-prevalence in controls.


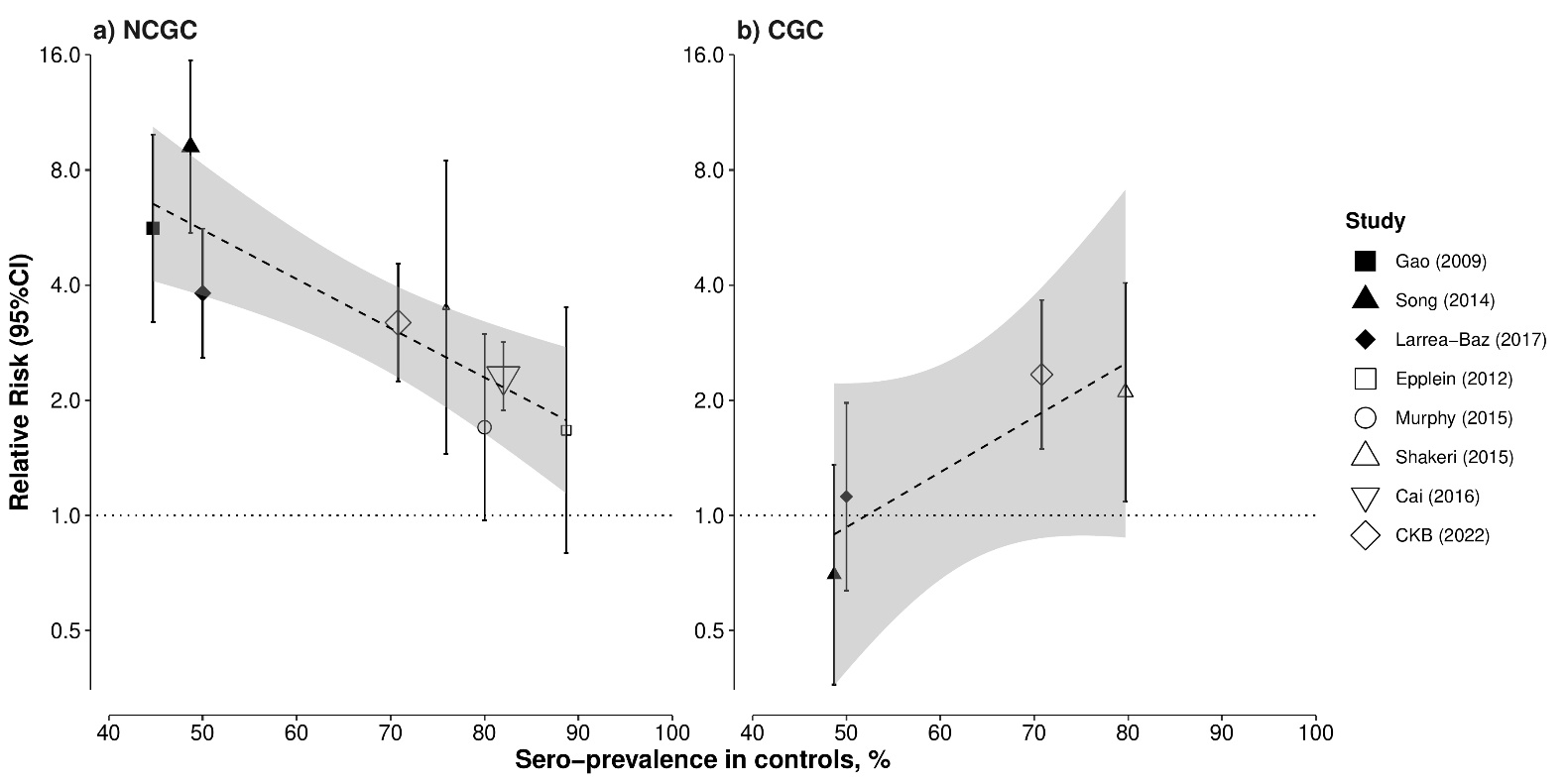


### Figure S7. Funnel plot of *H. pylori* CagA sero-positivity associated with a) non-cardia and b) cardia gastric cancer in meta-analysis of CKB and 7 published studies


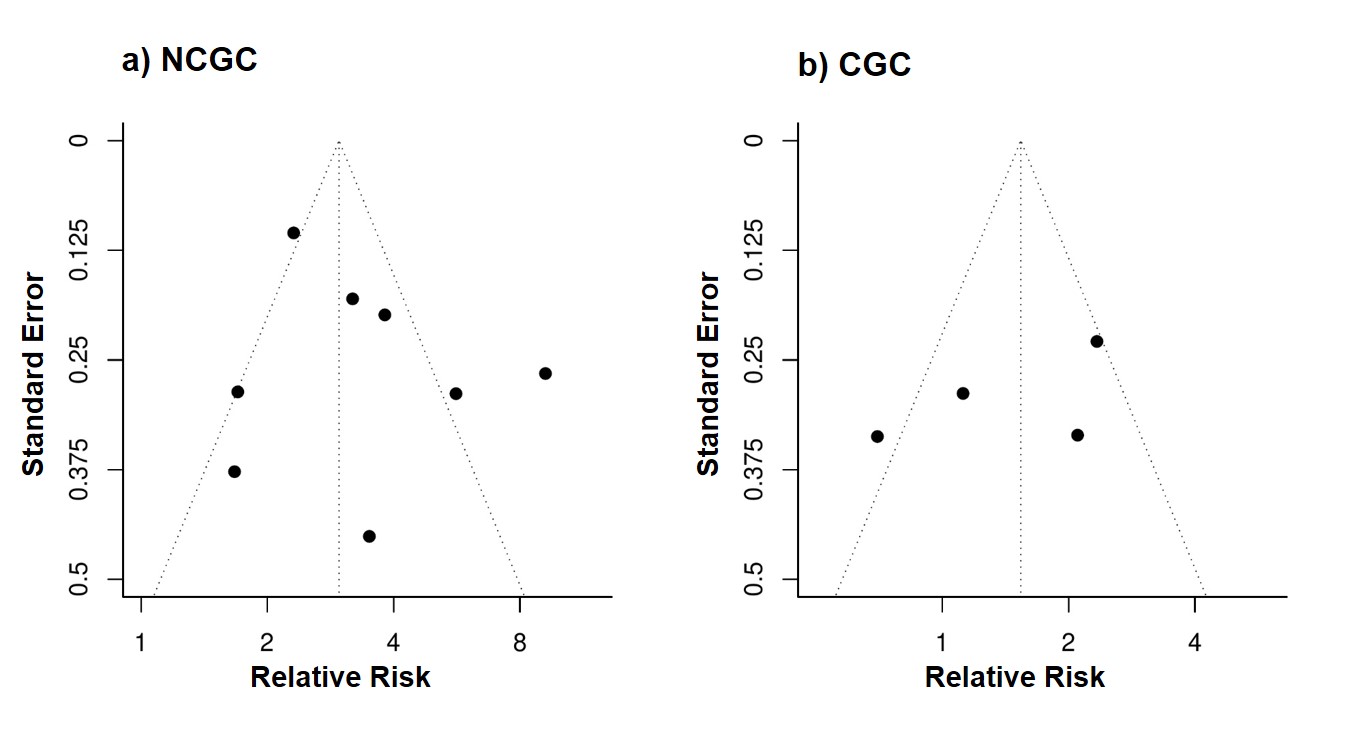


### Figure S8. Meta-analysis of CKB and other prospective studies of *H. pylori* HP1564 sero-positivity associated with risk of a) non-cardia and b) cardia gastric cancer

The size of the squares is inversely proportional to the variance of the logRR in each study. The error bars indicate the 95% CI. The dotted vertical line indicates the overall RR, and black diamond indicates it and its 95% CI.


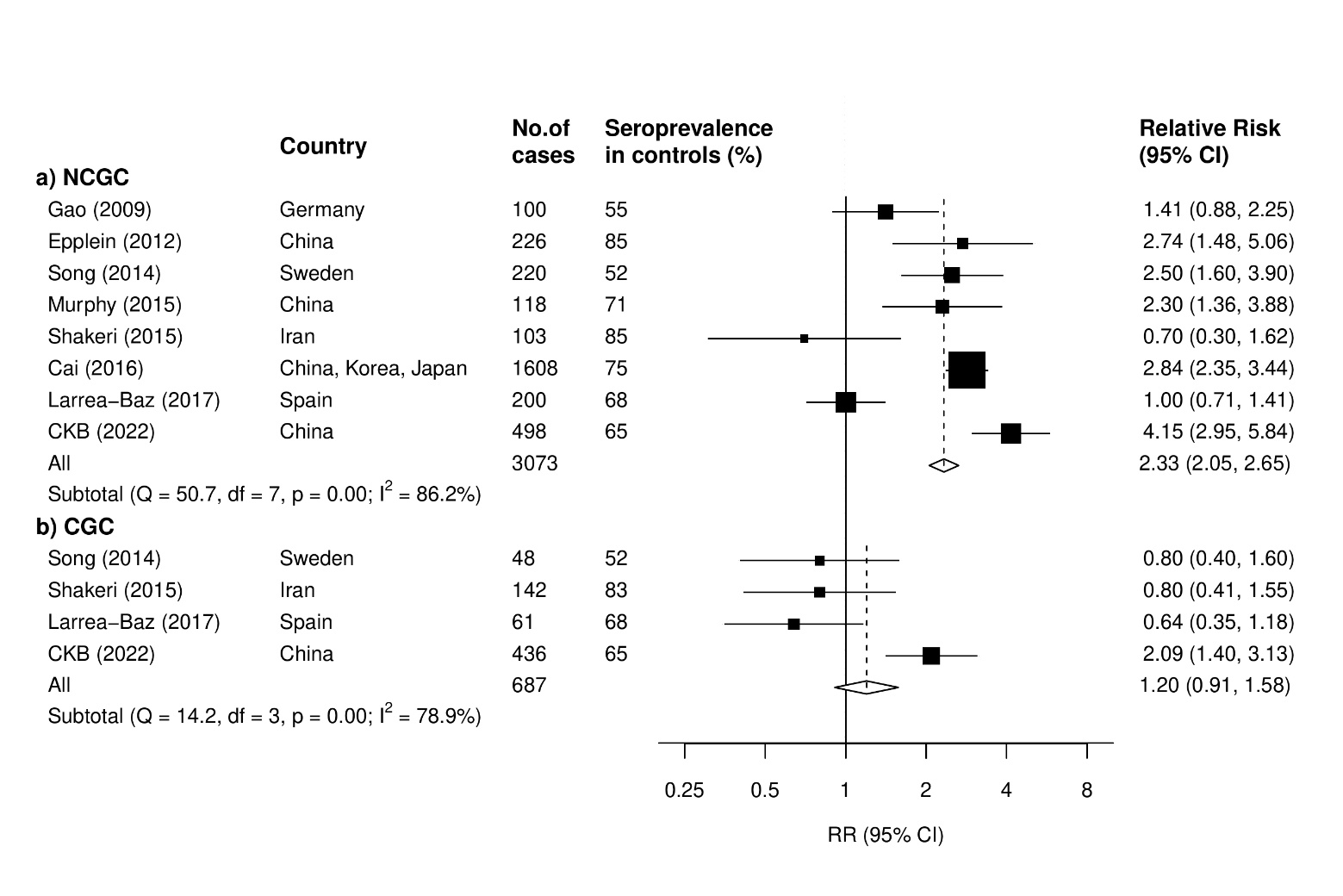


### Figure S9. Meta-analysis of CKB and other prospective studies of *H. pylori* GroEL sero-positivity associated with risk of a) non-cardia and b) cardia gastric cancer

The size of the squares is inversely proportional to the variance of the logRR in each study. The error bars indicate the 95% CI. The dotted vertical line indicates the overall RR, and open diamond indicates it and its 95% CI.


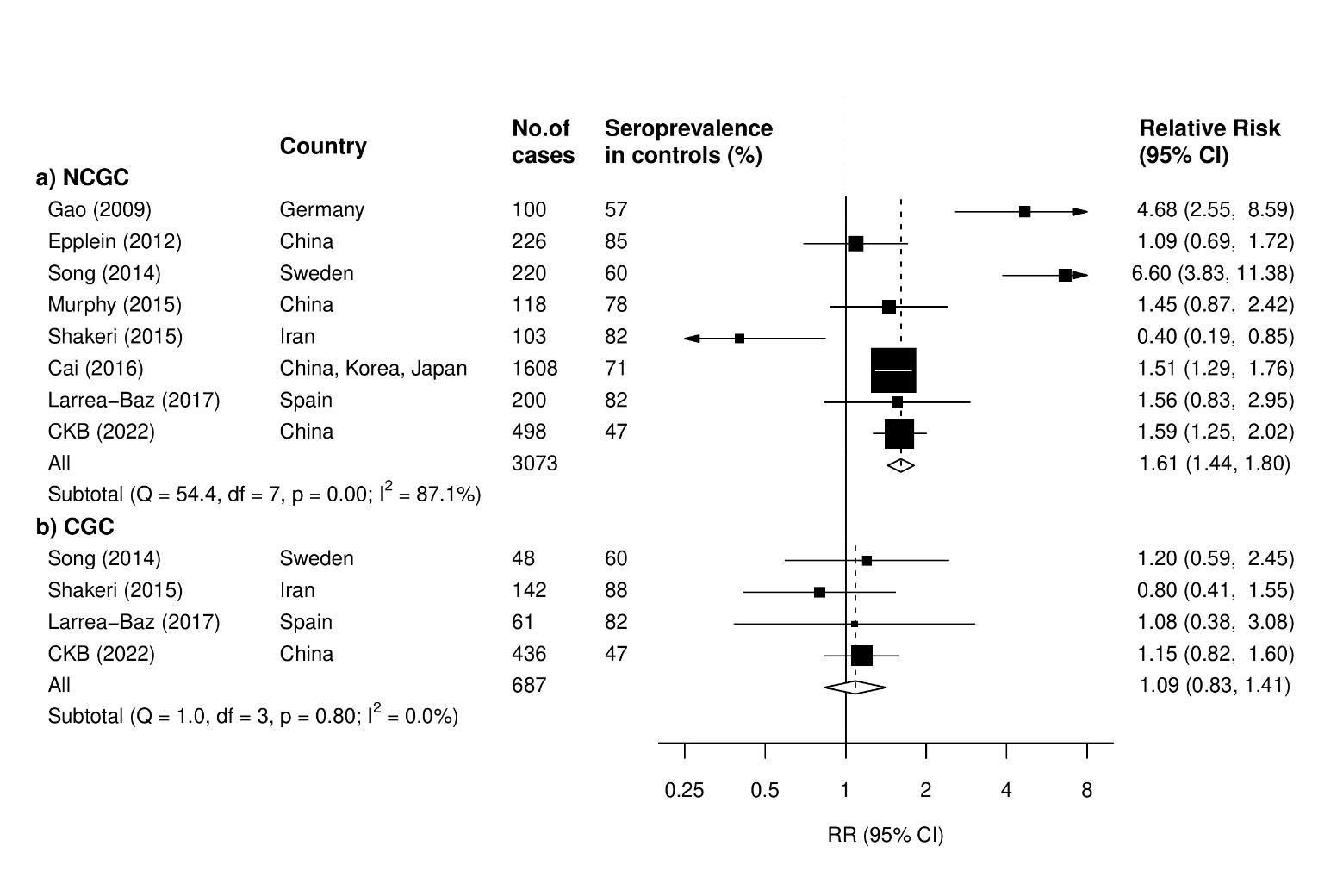


### Figure S10. Meta-analysis of CKB and other prospective studies of *H. pylori* HcpC sero-positivity associated with risk of a) non-cardia and b) cardia gastric cancer

The size of the squares is inversely proportional to the variance of the logRR in each study. The error bars indicate the 95% CI. The dotted vertical line indicates the overall RR, and black diamond indicates it and its 95% CI.


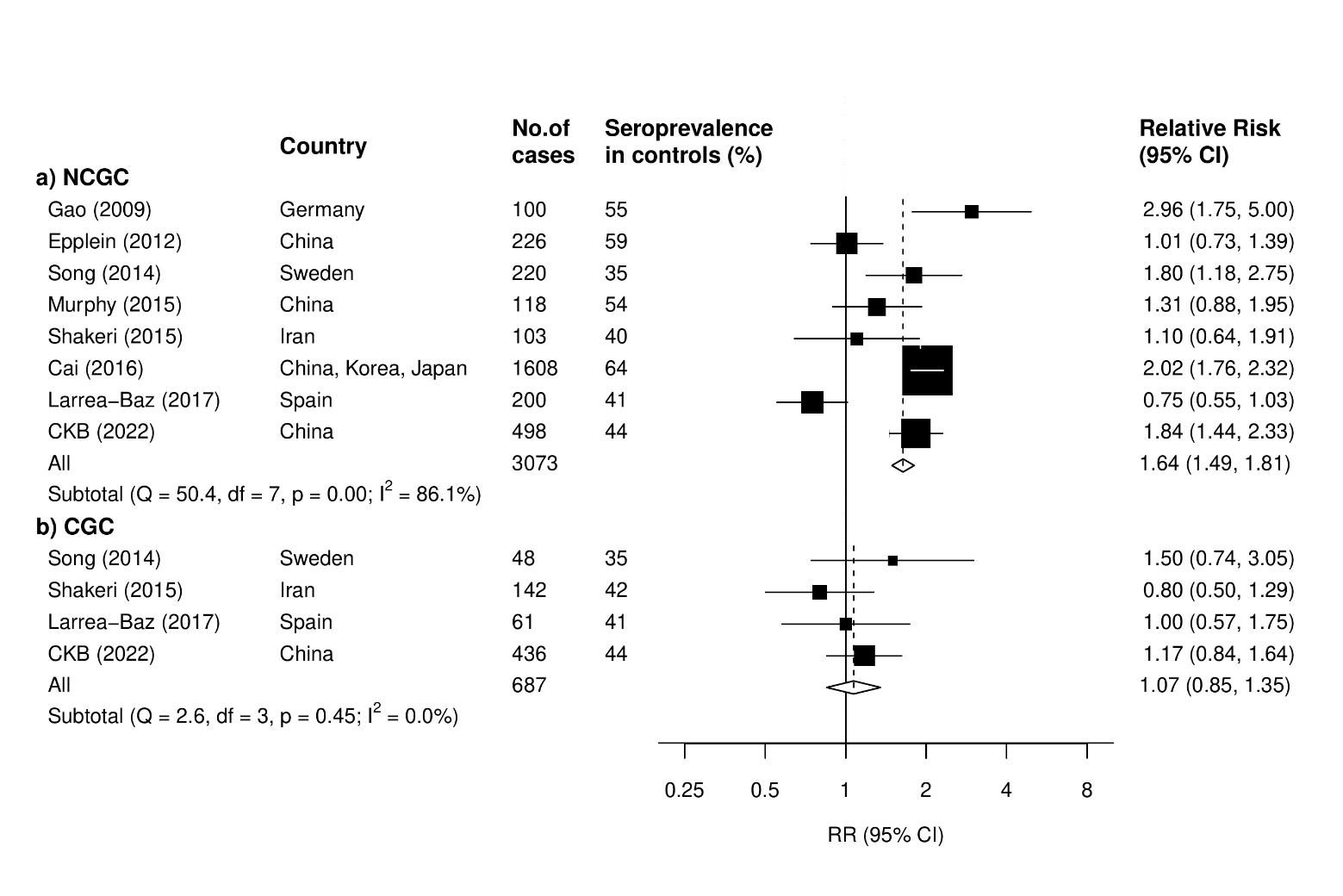


### Figure S11. Meta-analysis of CKB and other prospective studies of *H. pylori* VacA sero-positivity associated with risk of a) non-cardia and b) cardia gastric cancer

The size of the squares is inversely proportional to the variance of the logRR in each study. The error bars indicate the 95% CI. The dotted vertical line indicates the overall RR, and black diamond indicates it and its 95% CI.


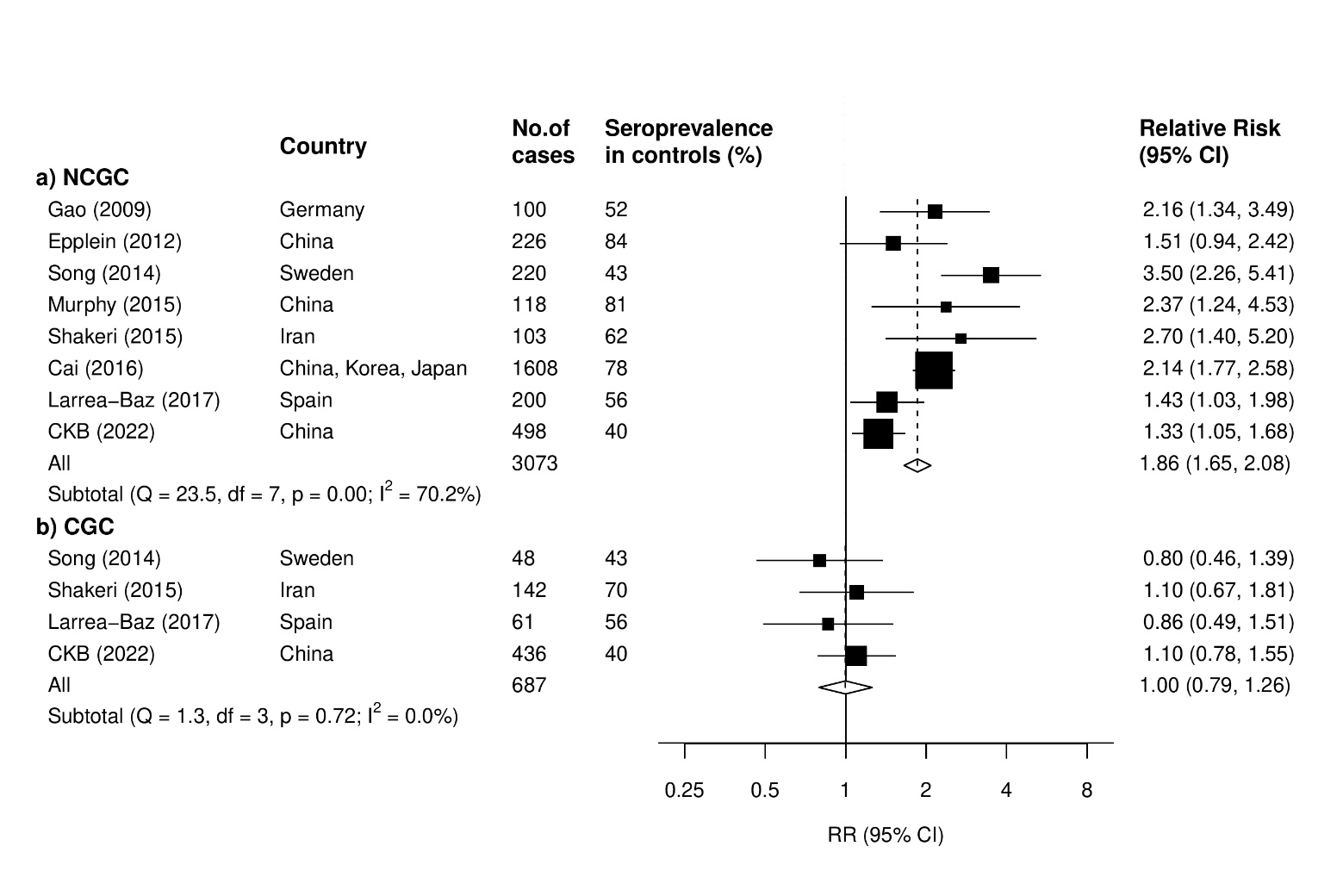


### Figure S12. Meta-analysis of CKB and other prospective studies of *H. pylori* HP0305 sero-positivity associated with risk of a) non-cardia and b) cardia gastric cancer

The size of the squares is inversely proportional to the variance of the logRR in each study. The error bars indicate the 95% CI. The dotted vertical line indicates the overall RR, and black diamond indicates it and its 95% CI.


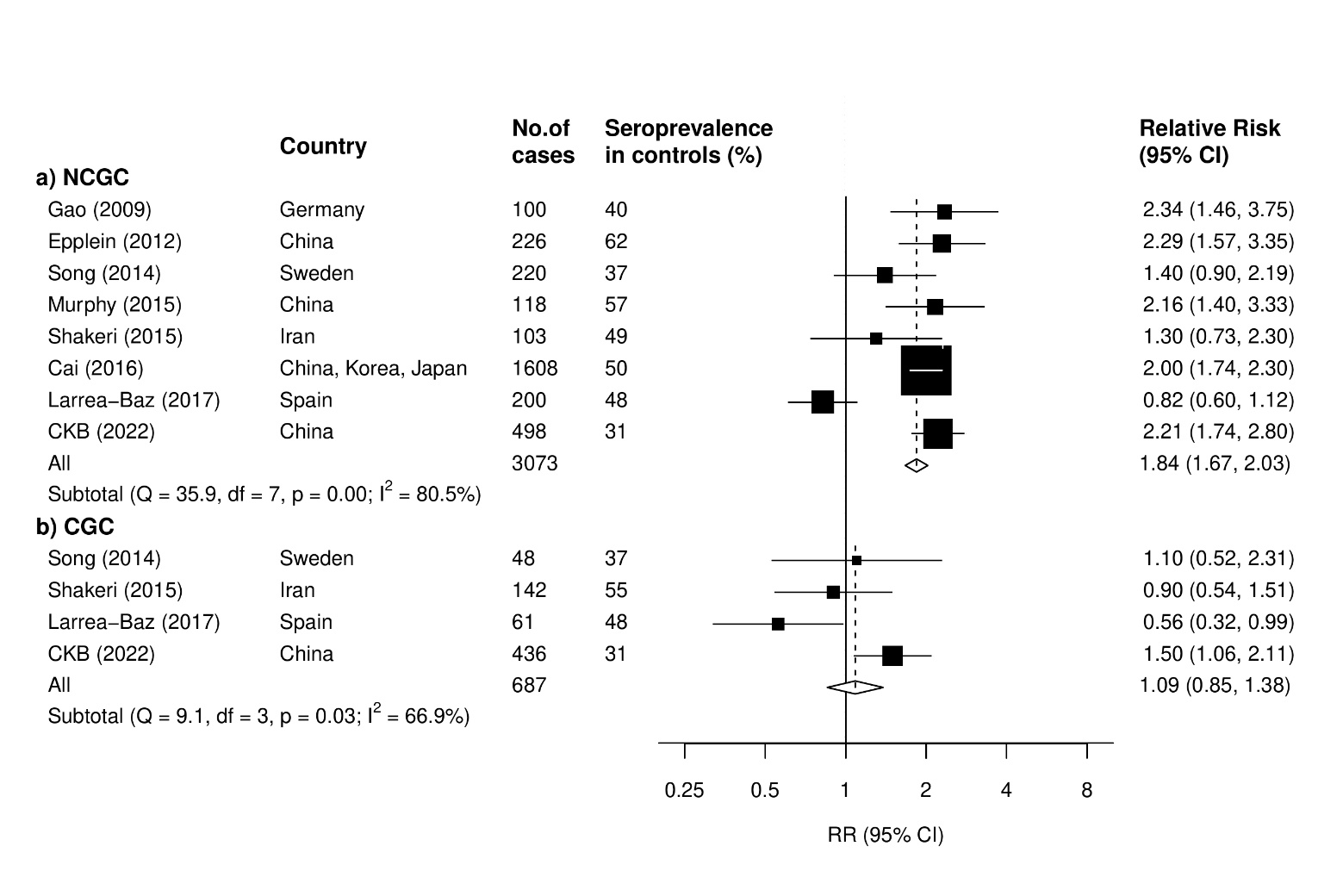


### Figure S13. Meta-analysis of CKB and other prospective studies of *H. pylori* NapA sero-positivity associated with risk of a) non-cardia and b) cardia gastric cancer

The size of the squares is inversely proportional to the variance of the logRR in each study. The error bars indicate the 95% CI. The dotted vertical line indicates the overall RR, and black diamond indicates it and its 95% CI.


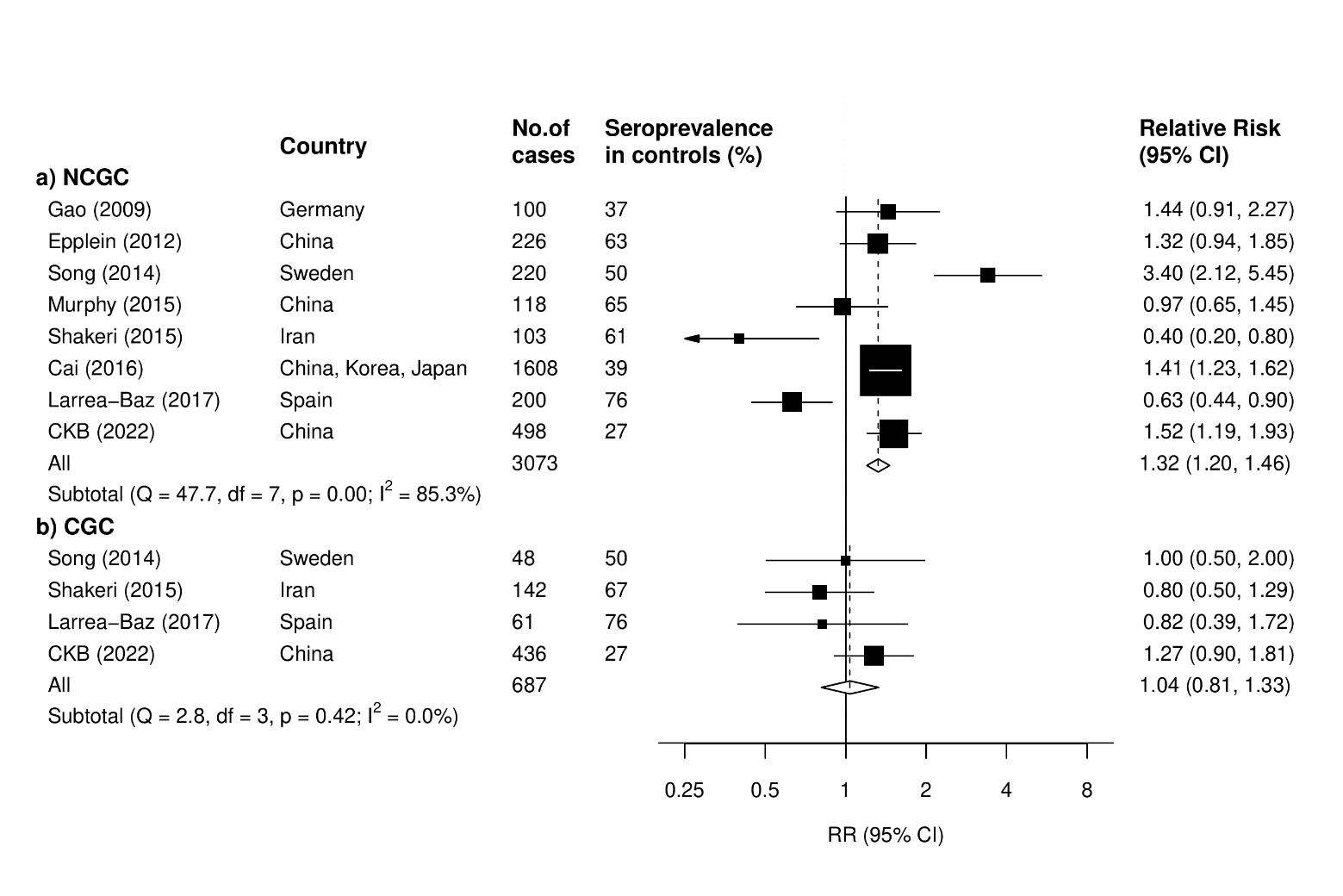


### Figure S14. Meta-analysis of CKB and other prospective studies of *H. pylori* Catalase sero-positivity associated with risk of a) non-cardia and b) cardia gastric cancer

The size of the squares is inversely proportional to the variance of the logRR in each study. The error bars indicate the 95% CI. The dotted vertical line indicates the overall RR, and black diamond indicates it and its 95% CI.

**
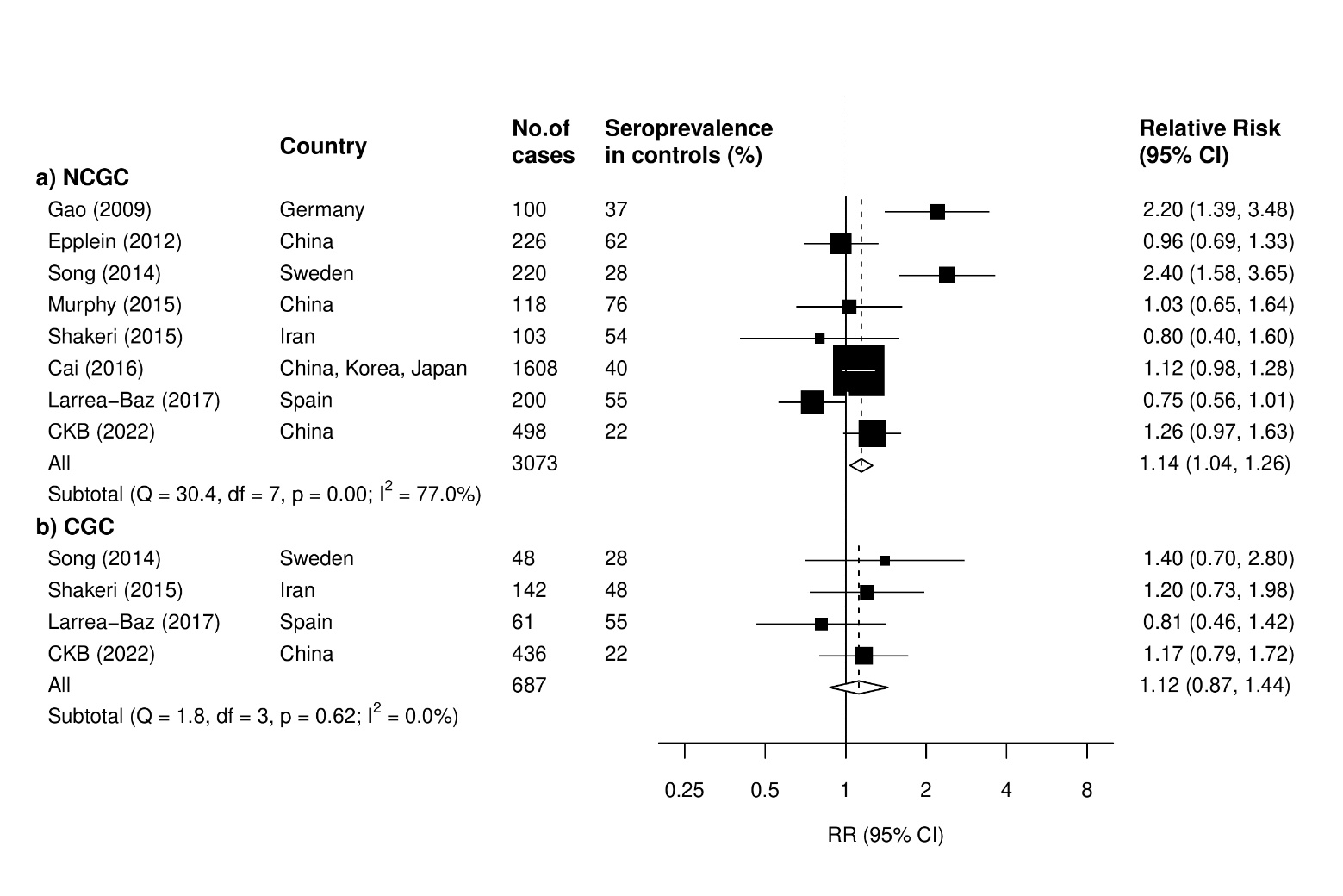
**

### Figure S15. Meta-analysis of CKB and other prospective studies of *H. pylori* Urease A sero-positivity associated with risk of a) non-cardia and b) cardia gastric cancer

The size of the squares is inversely proportional to the variance of the logRR in each study. The error bars indicate the 95% CI. The dotted vertical line indicates the overall RR, and black diamond indicates it and its 95% CI.

**
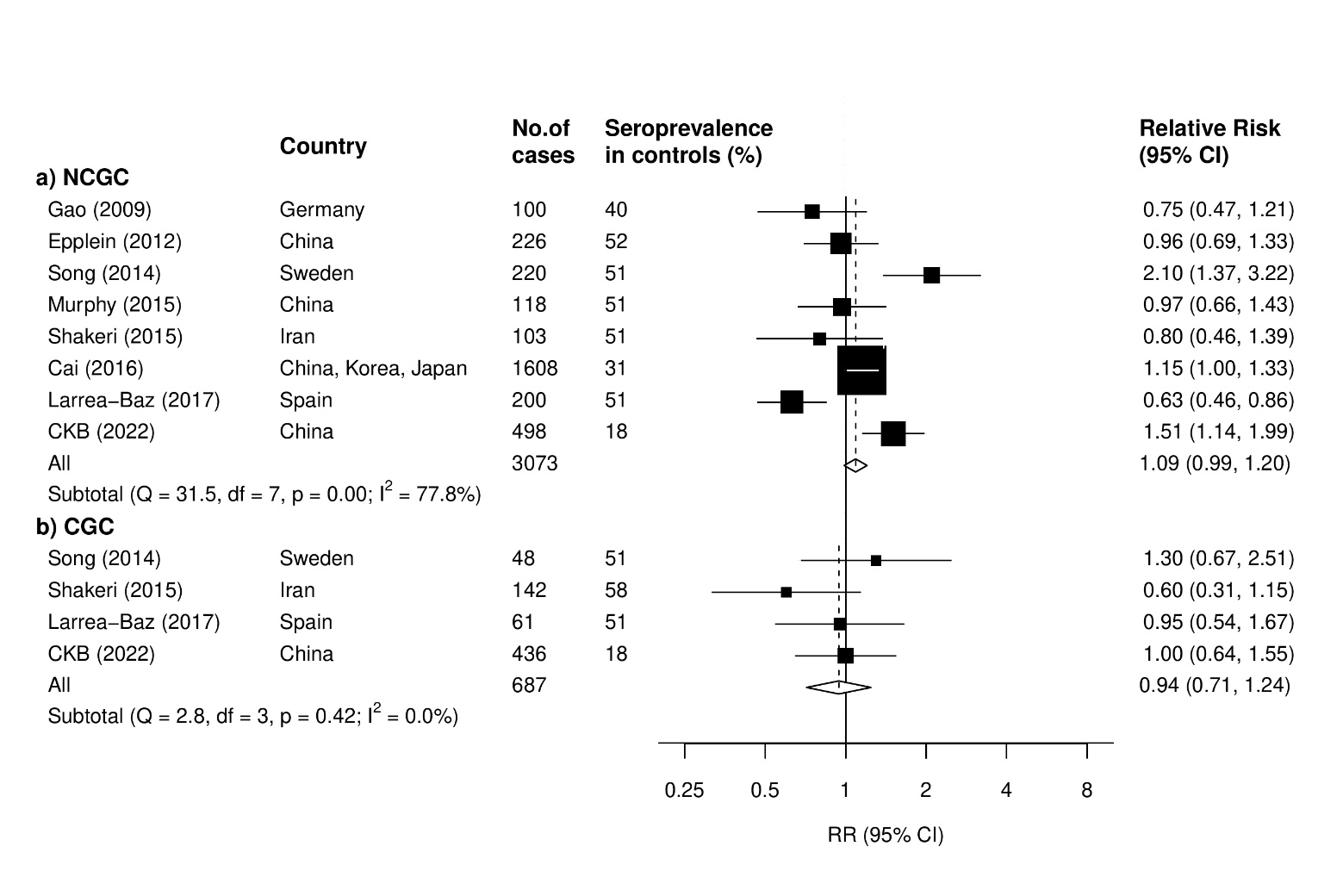
**

### Figure S16. Meta-analysis of CKB and other prospective studies of *H. pylori* HyuA sero-positivity associated with risk of a) non-cardia and b) cardia gastric cancer

The size of the squares is inversely proportional to the variance of the logRR in each study. The error bars indicate the 95% CI. The dotted vertical line indicates the overall RR, and black diamond indicates it and its 95% CI.


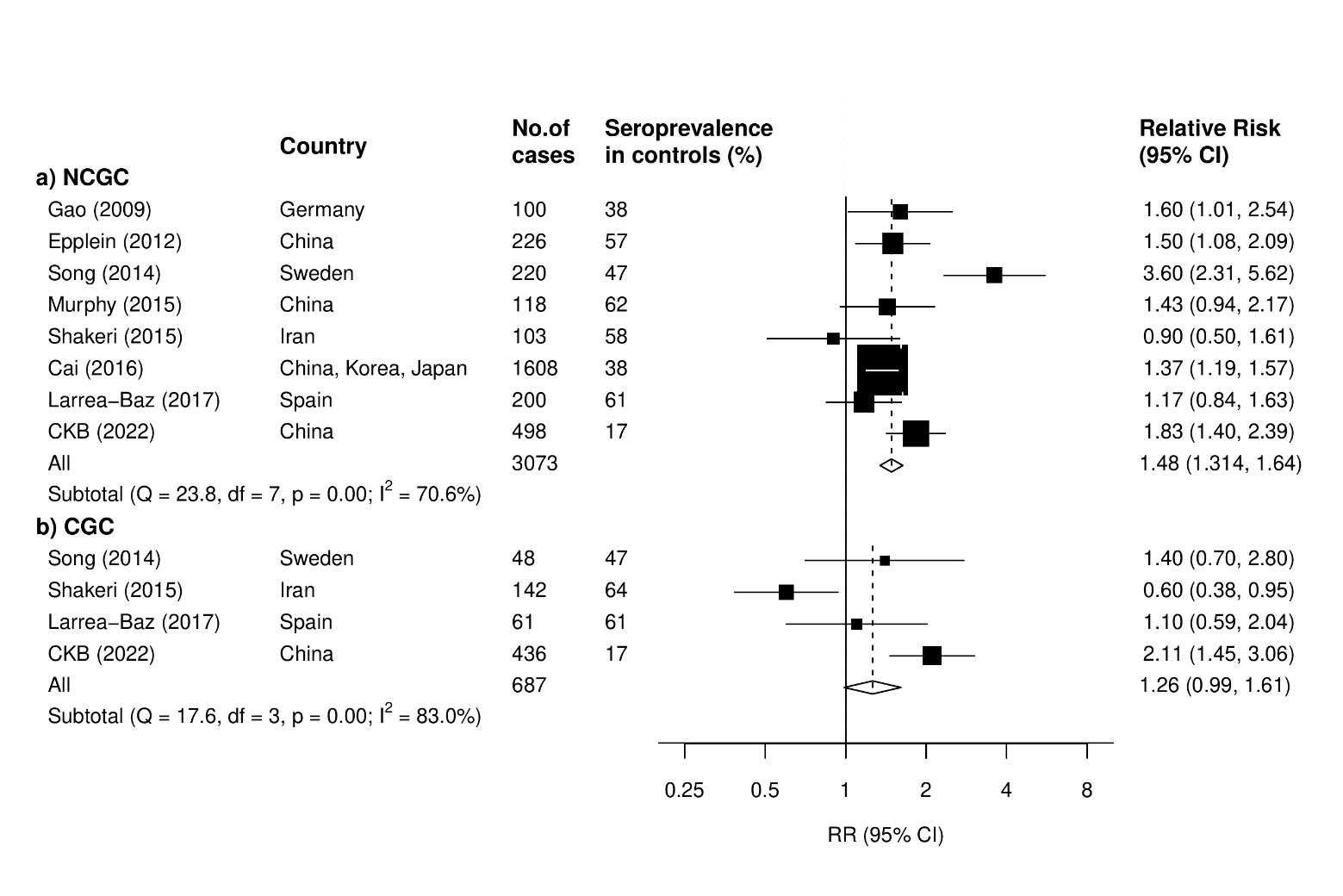


### Figure S17. Meta-analysis of CKB and other prospective studies of *H. pylori* Cad sero-positivity associated with risk of a) non-cardia and b) cardia gastric cancer

The size of the squares is inversely proportional to the variance of the logRR in each study. The error bars indicate the 95% CI. The dotted vertical line indicates the overall RR, and black diamond indicates it and its 95% CI.


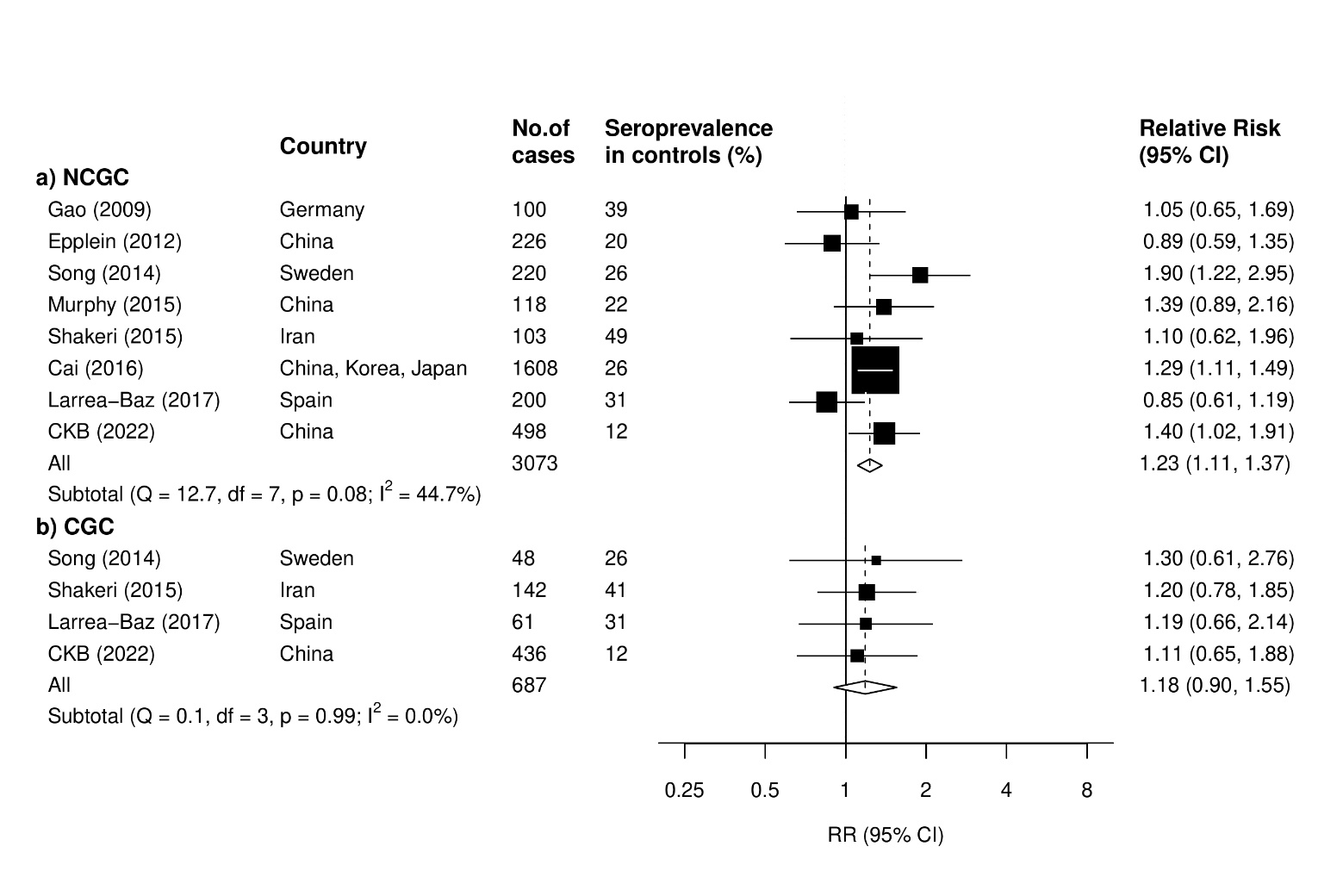


### Figure S18. Meta-analysis of CKB and other prospective studies of *H. pylori* HpaA sero-positivity associated with risk of a) non-cardia and b) cardia gastric cancer

The size of the squares is inversely proportional to the variance of the logRR in each study. The error bars indicate the 95% CI. The dotted vertical line indicates the overall RR, and black diamond indicates it and its 95% CI.


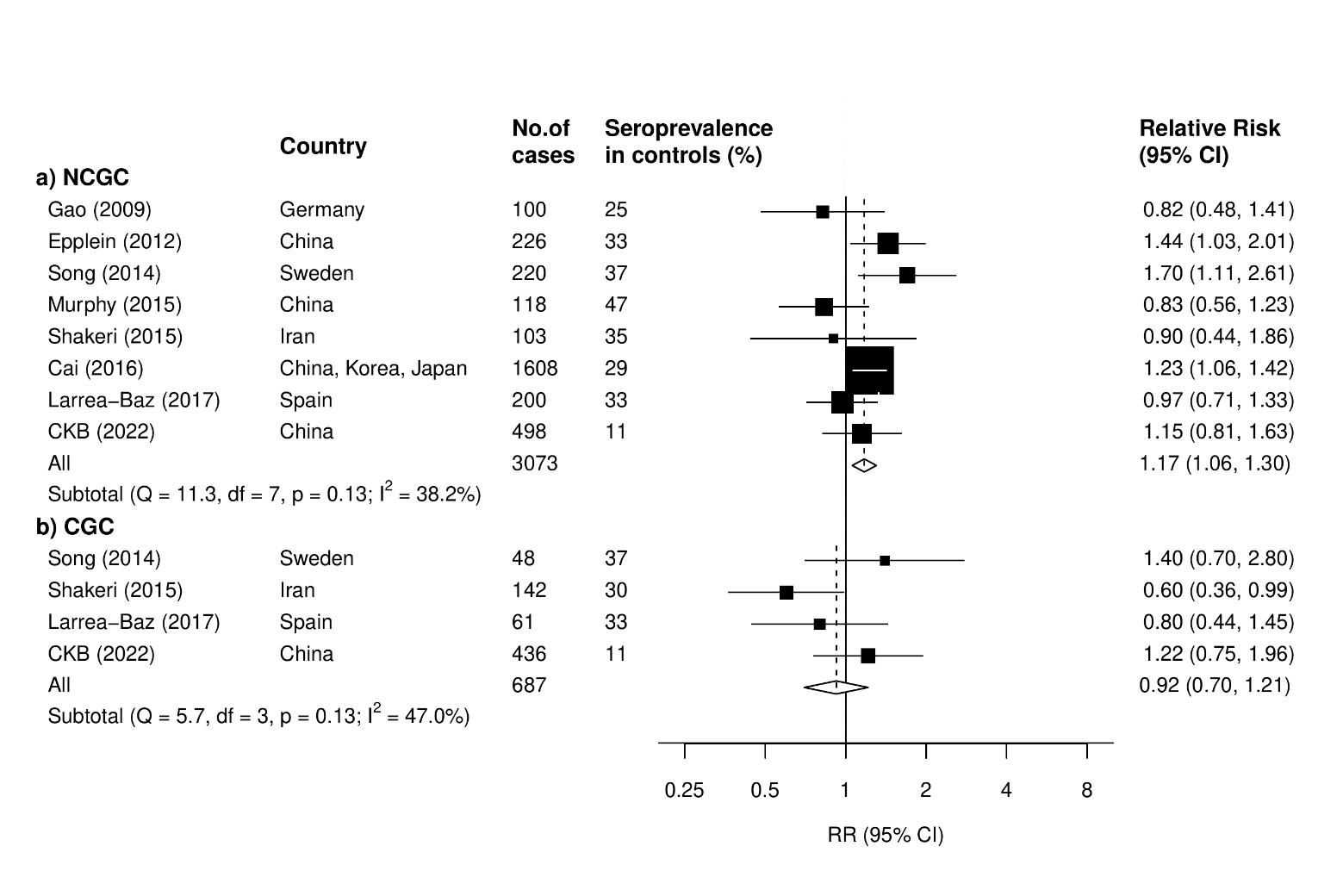


### Figure S19. Adjusted RRs for a) non-cardia and b) cardia gastric cancer associated with sero-positivity for 12 *H. pylori* antigens in a meta-analysis of the CKB and 6 published studies, by regions

Solid boxes represent the Relative Risks (RRs) associated with sero-positivity of *H. pylori* antigen for studies from different regions, with their size inversely proportional to the variance of the logRR. Diamonds represent summary RRs for all regions. For each antigen, there are 3 studies in Europe (520 cases) and 4 studies in Asia (2450 cases) for non-cardia GC, 2 studies in Europe (109 cases) and 1 studies in Asia (436 cases) for cardia GC, excluding the study from Iran.


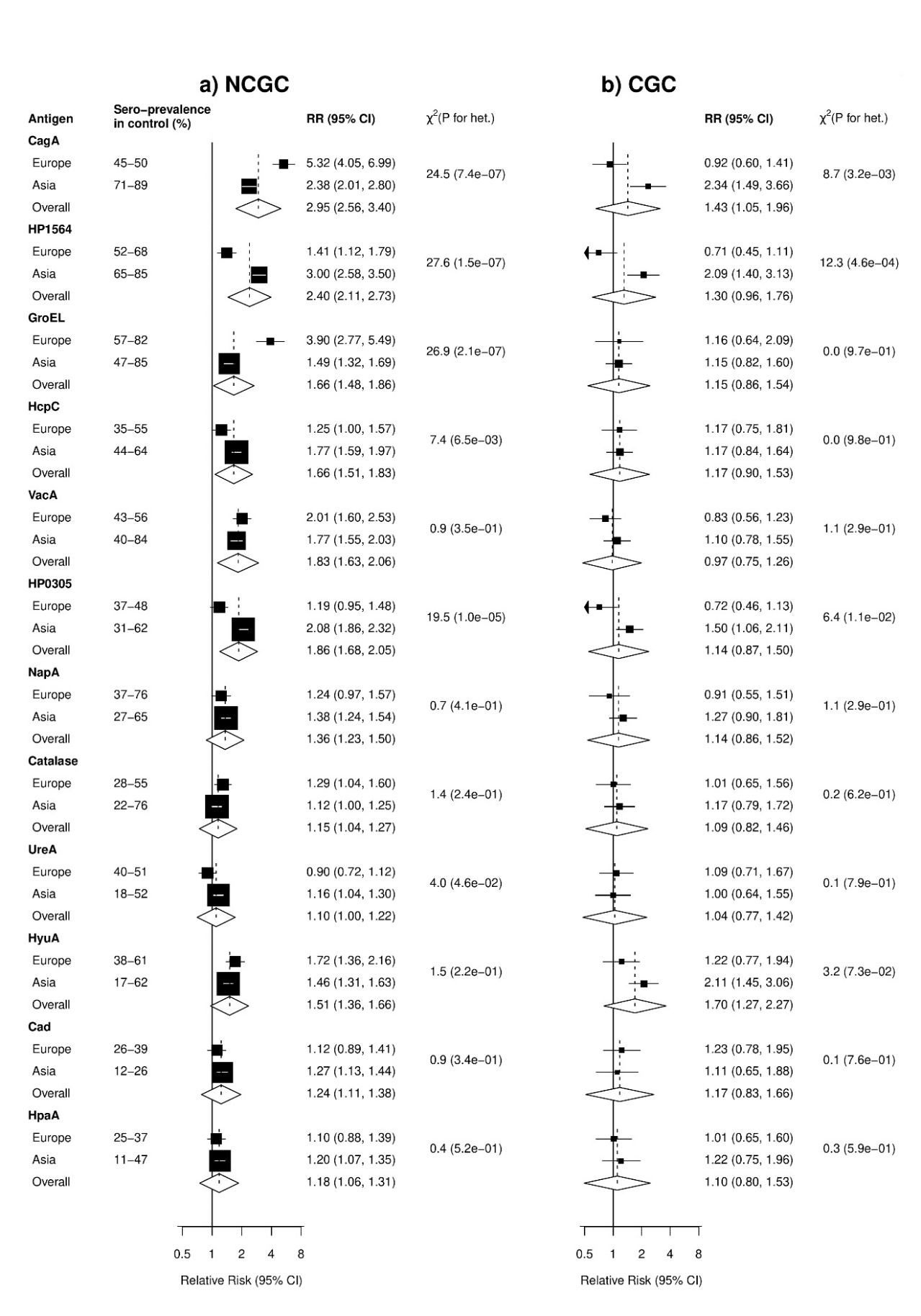

Supplement: dyad007_Supplementary_Data [file dyad007_supplementary_data.docx]
